# Replication landscape of the human genome

**DOI:** 10.1038/ncomms10208

**Published:** 2016-01-11

**Authors:** Nataliya Petryk, Malik Kahli, Yves d'Aubenton-Carafa, Yan Jaszczyszyn, Yimin Shen, Maud Silvain, Claude Thermes, Chun-Long Chen, Olivier Hyrien

**Affiliations:** 1 Ecole Normale Supérieure, Institut de Biologie de l'ENS (IBENS), and Inserm U1024, and CNRS UMR 8197, 46 rue d'Ulm, Paris F-75005, France; 2 Institute for Integrative Biology of the Cell (I2BC), CEA, CNRS, Université Paris-Sud, UMR 9198, FRC 3115, Avenue de la Terrasse, Bâtiment 24, Gif-sur-Yvette, Paris F-91198, France

## Abstract

Despite intense investigation, human replication origins and termini remain elusive. Existing data have shown strong discrepancies. Here we sequenced highly purified Okazaki fragments from two cell types and, for the first time, quantitated replication fork directionality and delineated initiation and termination zones genome-wide. Replication initiates stochastically, primarily within non-transcribed, broad (up to 150 kb) zones that often abut transcribed genes, and terminates dispersively between them. Replication fork progression is significantly co-oriented with the transcription. Initiation and termination zones are frequently contiguous, sometimes separated by regions of unidirectional replication. Initiation zones are enriched in open chromatin and enhancer marks, even when not flanked by genes, and often border ‘topologically associating domains' (TADs). Initiation zones are enriched in origin recognition complex (ORC)-binding sites and better align to origins previously mapped using bubble-trap than λ-exonuclease. This novel panorama of replication reveals how chromatin and transcription modulate the initiation process to create cell-type-specific replication programs.

## Abstract (web summary)

The physical origin and termination sites of DNA replication in human cells have remained elusive. Here the authors use Okazaki fragment sequencing to reveal global replication patterns and show how chromatin and transcription modulate the process.

## Graphical abstract

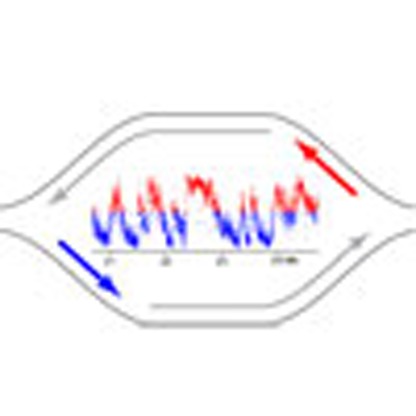

Genome-wide identification of mammalian replication origins^1^,^2^,^3^ has been attempted by various methods including trapping of replication-bubbles^4^,^5^, purification of RNA-primed single DNA strands^6^,^7^,^8^,^9^,^10^,^11^, immunoprecipitation of BrdU-labelled DNA^12^ or chromatin immunoprecipitation of origin recognition complex (ORC)^13^, followed by microarray hybridization or high-throughput sequencing. The number and position of origins thus detected present discrepancies^1^,^3^ (from 12,000 (ref. 4) to 250,000 (ref. 9) putative origins detected genome-wide). In addition, these methods cannot precisely quantify origin efficiency nor map termination.

An orthogonal method to map replication is based on the analysis of nucleotide compositional asymmetries of the two DNA strands^1^,^14^,^15^. In most bacterial genomes, the GC and TA skews *S*_GC_=(*G−C*)/(*G+C*) and *S*_TA_=(*T−A*)/(*T+A*) are strictly correlated with replication direction, suggesting that mutational asymmetry of the leading and lagging strands led to skew accumulation over evolutionary times^14^. Thus, detection of an abrupt sign inversion of the skew is a standard tool to predict origin and termini in bacteria^16^. Upward skew jumps similar to bacterial origins have been detected at 1,546 sites in the human genome^17^,^18^. Between jumps, the skew decreased in a linear manner, suggesting a progressive inversion of the average direction of replication (in germline cells) across N-shaped domains of skew, perhaps due to the random termination between the two origins^19^. When compared with somatic cell replication timing profiles^20^,^21^,^22^, skew N-domains indeed coincided with U-shaped domains of replication timing, with borders (skew jumps) replicating early and centres replicating later^15^,^21^,^23^,^24^,^25^. However, detectable N/U-domains together cover only one-third to one-half of the human genome and it is unclear if replication of the remainder follows similar rules. Furthermore, N/U-domains are much longer (0.5–3 Mb) than typical inter-origin distances (100–200 kb) suggesting that additional intradomain origins may fire in a sequential pattern from N/U-domain borders to centres^15^,^25^,^26^. The low resolution of skew and replication timing profiles precluded precise location of initiation and termination events along N/U-domains.

A novel method that permits a high-resolution, quantitative analysis of replication fork initiation, progression and termination has been recently developed in *Saccharomyces cerevisiae*^27^,^28^. The principle is to isolate and sequence Okazaki fragments to reveal the proportions of rightward- (*R*) and leftward- (*L*) moving forks throughout the genome. Zones of predominant initiation (termination) can then be detected as upward (downward) slopes in replication fork directionality (RFD=*R−L*) profiles (Fig. 1a–c). Since the very low amount of Okazaki fragments present in wild-type cells represents a challenge, these studies used conditional lethal mutants that massively increase Okazaki fragment abundance^27^,^28^. The results were consistent with preexisting origin identifications and allowed to estimate origin efficiency. Furthermore, the results demonstrated that a large fraction of the genome can be replicated in either orientation and that termination occurs between origins at positions dictated by their relative firing times.

Here we present a new methodology to isolate and sequence Okazaki fragments (OK-Seq) that does not require overproducer mutant cells. This allowed us to determine RFD genome-wide in asynchronous cultures of two human cell lines, without any genetic modification. We infer the location of replication initiation and termination events at high resolution and paint a novel, comprehensive landscape of human genome replication in these two cell types.

We validate the replication model of N/U-domains. We identify in the two cell lines 9,386 and 5,684 (4,150 shared) broad initiation zones that typically support a single but randomly located initiation event. These zones are mostly non-transcribed, often but not always circumscribed by active genes, generally enriched in open chromatin and enhancer epigenetic marks, and often located at TAD borders. Initiation zones associated and not associated with active genes fire early and late in S phase, respectively. Replication terminates very dispersively between initiation zones, either within transcribed, early-replicating gene bodies or within large, late-replicating, silent DNA regions. A significant though incomplete co-orientation of replication with transcription is observed, due to preferential (but not exclusive) initiation of DNA replication upstream of active genes. The number of initiation zones detected in this work accounts for only a fraction of total initiation events. We, therefore, suggest that the human genome undergoes a superposition of efficient initiation at circumscribed ‘master' zones identified here and more dispersed, less efficient initiation elsewhere.

## Results

### Genome-wide profiling of RFD

**Figure 2:**
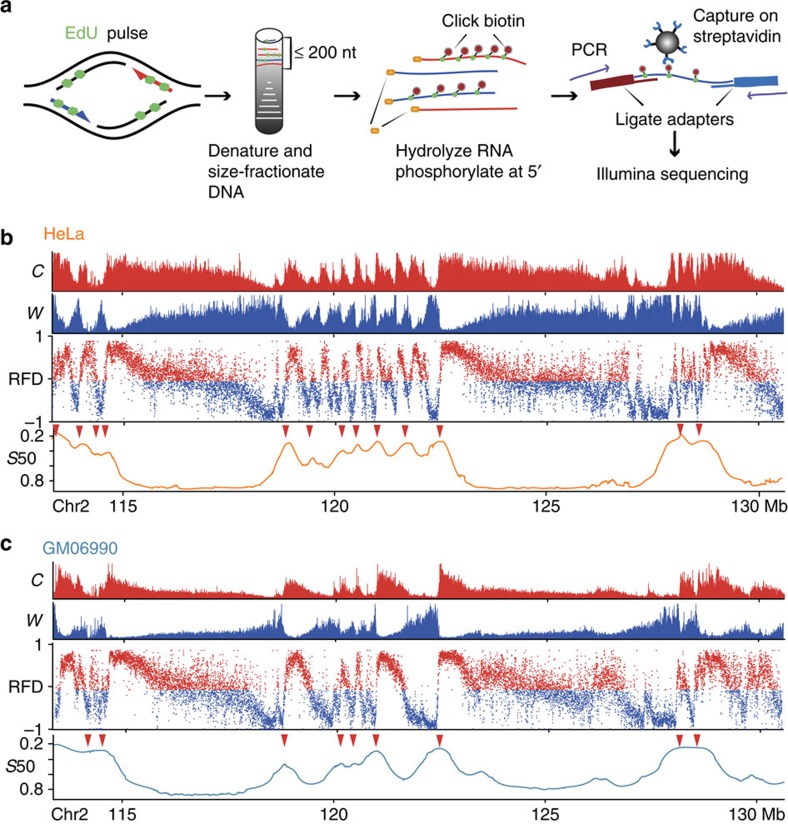
Replication directionality revealed by OK-Seq. (**a**) Okazaki fragment purification scheme (see text and Methods section). (**b**,**c**) Exemplary HeLa (**b**) and GM06990 (**c**) profiles. Top, Okazaki fragments mapping to Crick (C, red) or Watson (W, blue) strands. Middle, RFD computed in 1 kb adjacent windows. Bottom, replication timing profile (*S*_50_; see Methods section); red arrows, early peaks (*S*_50_<0.5).

Our methodology is based on the replicative incorporation of the thymidine analogue 5-ethynyl-2′-deoxyuridine (EdU) followed by purification and sequencing of labelled Okazaki fragments (Fig. 2a and Methods section). Okazaki fragments purified from two cell types, HeLa (adenocarcinoma) and GM06990 (lymphoblastoid) revealed a totally new, full panorama of replication fork progression along the genome with excellent reproducibility (Fig. 2b,c, Supplementary Fig. 1). RFD profiles displayed series of alternating quasi-linear ascending, descending and flat segments (AS, DS, FS) of varying size and slope. RFD often reached values >0.9 or <−0.9, indicating nearly complete purity of Okazaki fragments. A small-scale (<300 kb) saw-tooth pattern was mostly observed in early-replicating regions (for example, 118–123 Mb, Fig. 2b,c). By contrast, most late-replicating regions displayed megabase-sized DS and/or FS of low absolute values of RFD (|RFD|) (for example, 115–118 Mb, Fig. 2b,c).

### OK-Seq validates replication model of N/U domains

**Figure 3:**
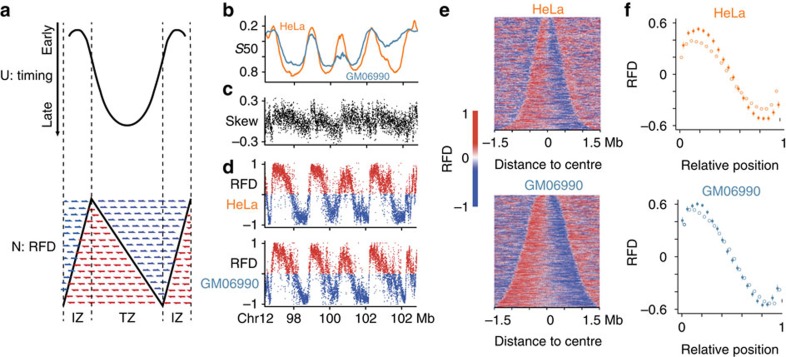
OK-Seq corroborates the replicative organization of N/U-domains. (**a**) U-domains of replication timing (top) are predicted to show an N-shaped RFD profile (bottom). The steep slopes at U-domain borders predict a high |RFD| whereas the flat slope at the central, late-replicating valley predicts a null RFD^15^,^25^,^26^. Blue and red arrows, expected proportions of Okazaki fragments from either strand across zones of predominant initiation (IZ) at borders and predominant termination (TZ) at center of U-domain. Dark line, expected RFD profile. The mathematical relationship *d*MRT*/dx*=RFD*/v* implies that RFD increases (AS) when the timing profile is convex (*d*^2^MRT*/dx*^2^ >0) and decreases (DS) when it is concave (*d*^2^MRT*/dx*^2^<0; note that the time axis is oriented from top to bottom). (**b**) HeLa (orange) and GM06990 (teal blue) replication timing profiles showing four adjacent U-domains^25^; (**c**) nucleotide compositional skew profile (see Methods section) showing skew N-domains^19^ matching replication timing U-domains; (**d**) N-shaped RFD profiles determined by OK-Seq matching N-domains. (**e**) Heatmap of RFD along the 1,410 HeLa and 878 GM06990 U-domains^25^, ordered by length and centred at zero abscissa. (**f**) Mean RFD values (±s.e.m.) along rescaled U-domains (all domains, empty circles; domains matching (>80% overlap) N-domains, full circles).

Previously, we detected in these cells, independently of skew analysis, 900–1,500 megabase-scaled domains characterized by parabolic (U-shaped) replication timing profiles and we predicted they would display N-shaped RFD profiles (Fig. 3a,b). This prediction results from the mathematical relationship between mean replication timing (MRT), fork speed (*v*) and RFD: *d*MRT*/dx*=RFD*/v* (refs 25, 26, 29) (the derivative of a parabolic profile is a straight line). In words, assuming *v* is constant, the steep slopes at the borders of the U predict a high |RFD| whereas the flat slope at the center of the U predicts a null RFD. In addition, U-domains frequently coincided with independently detected N-shaped domains of nucleotide compositional skew profile^19^,^25^,^26^,^29^ (Fig. 3c), proposed to reflect an N-shaped pattern of germline RFD^19^,^24^. OK-Seq fully verified the predicted 1–2 Mb N-shaped RFD gradients of replication timing U-domains and skew N-domains (Fig. 3d–f). These linear gradients of RFD likely result from cascades of origin firing propagating from domain borders to center^15^,^25^,^26^. The remarkable similarity of the RFD gradients deduced from skew^19^ or replication time^25^ or determined by OK-Seq provides a strong reciprocal validation of these data. Moreover, the superior resolution of OK-Seq reveals replication initiation and termination zones with much higher precision.

### Replication initiates stochastically within broad zones

**Figure 4:**
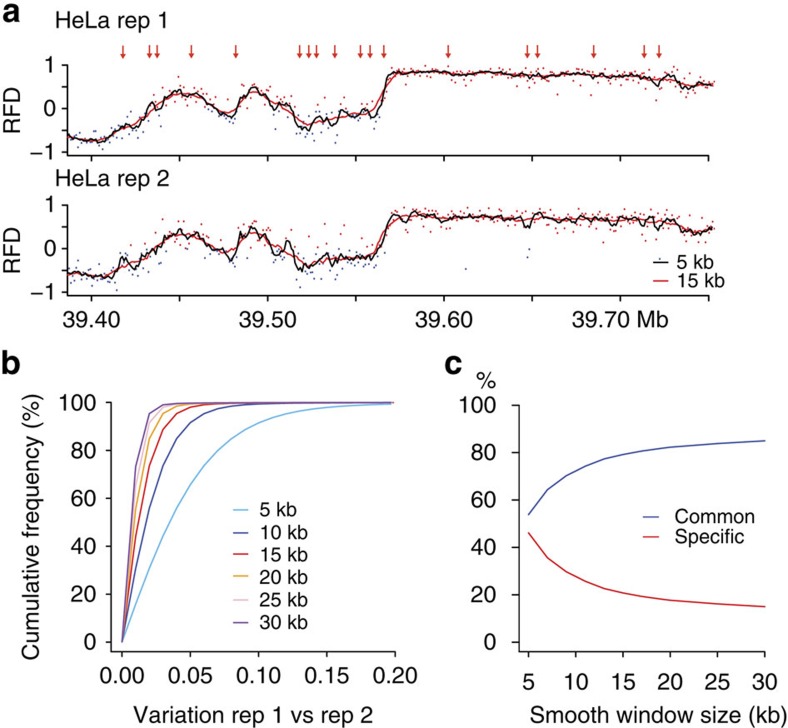
Effect of HMM sliding window on AS detection reproducibility between biological replicates. (**a**) RFD profiles corresponding to two OK-seq experiments (rep 1 and rep 2); RFD was computed in sliding windows of size *l*=5 kb (dark line) and *l*=15 kb (red line); at each point *n* of the RFD profiles, the slope was computed as *s*(*n*)=RFD_*n*+1_−RFD_*n*_ ; the difference ∂(*n*)=*s*_1_(*n*)−*s*_2_(*n*) between the slopes of the two profiles was computed along the genome; red arrows indicate the positions at which ∂(*n*)>0.10 in the case where *l*=5 kb. (**b**) ∂(*n*) was computed for various values of *l* (*l*=5, 10, 15, 20, 25, 30 kb); the cumulated proportion of ∂(*n*) (proportion of positions for which ∂(*n*)>*x*) was displayed for each *l* value. For *l*=5 kb, 40% of genome positions presented slopes differing by more than 0.05 (light blue). This proportion dropped down to 10 % for *l*=10 kb, and to 4% for *l*=15 kb. This showed that the difference between the two profiles strongly decreased for *l* ≥15 kb. (**c**), AS of the rep 1 and rep 2 profiles were determined using the HMM procedure (see Methods section) for various sizes of *l*. The proportion of AS common to both replicate profiles (blue) or specific to one of the two profiles (red) was displayed as a function of *l*. The proportion of AS specific to only one replicate, that is, that would not be considered as *bona fide* AS, decreased from almost 50% for *l*=5 kb to 20% for *l*=15kb and then decreased more slowly for larger *l* values. This means that analysis of RFD profiles with *l*<15 kb would lead to an excessive proportion of AS not conserved between replicates, likely corresponding to too small signal/noise values.

**Figure 5:**
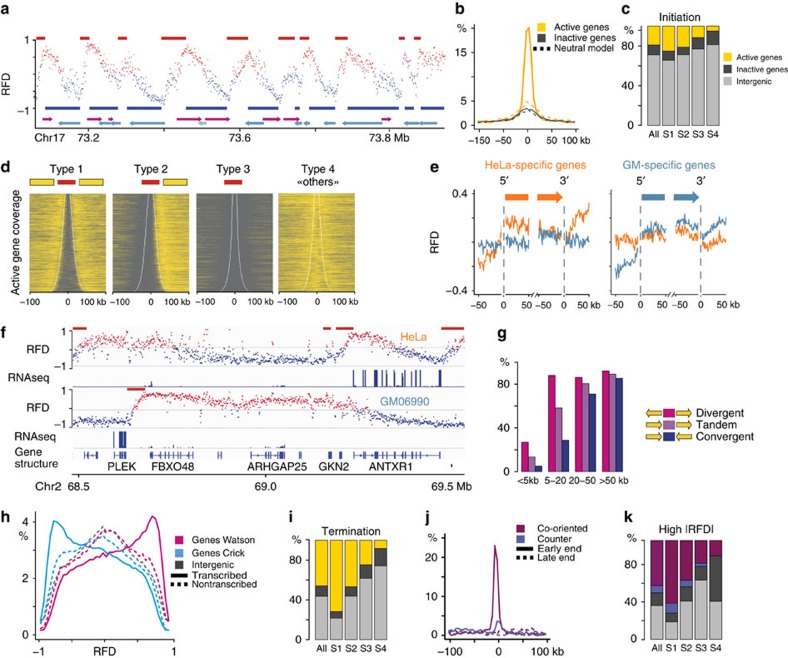
Transcription impact on replication landscape. (**a**) Red (blue) lines above (below) HeLa RFD profile indicate ascending (descending) HMM-detected segments (see Methods section); magenta and cyan arrows indicate genes. (**b**) Line, histogram of distance between an AS end and the nearest active (yellow) or inactive (charcoal) gene; dots, neutral model with randomly positioned AS (see Methods section). (**c**) Percentage of AS covering (majority rule) active genes (yellow), inactive genes (charcoal), intergenic regions (silver), shown for all AS or for AS in successive periods of S phase (S1–S4; see Methods section). (**d**) Heatmap of active genes around the four types of HeLa AS described in the text; yellow boxes, active genes flanking AS (red lines). (**e**) Mean RFD profiles of HeLa (orange) and GM06990 (blue) around 5′ and 3′ ends of isolated genes (see Methods section) expressed specifically in HeLa (left) or GM06990 (right). (**f**) Examples of AS associated with genes specifically expressed in HeLa (ANTXR1) or GM06990 (PLEK). (**g**) Proportions of segments between active genes, which overlap (≥1 nt) an AS, for different distances and orientations of flanking genes (HeLa). (**h**), histograms of HeLa RFD for intergenes (grey), genes oriented rightward (magenta) or leftward (cyan), transcribed (lines) or not (dash). (**i**) Percentage of HeLa DS covering genes or not, displayed as in **c**. (**j**) Histogram of distance between early- (line) or late- (dash) replicating end of high |RFD| (|RFD|>0.6) FS and 5′ or 3′ end of the nearest co-oriented (eggplant) or counter-oriented (blue) active gene. (**k**) Percentage of HeLa high |RFD| FS covered (majority rule) by sequences from co-oriented (eggplant) or counter-oriented (blue) active genes, inactive genes (charcoal), intergenic regions (silver).

We used a hidden Markov model (HMM) to detect zones of predominant initiation as AS (see Methods section). The RFD values were computed within 15 kb sliding windows (stepped by 1 kb) to ensure reproducible AS detection (Fig. 4). In HeLa and GM06990, we detected 9,836 and 5,684 AS (mean size 30 kb, range 6–150 kb), covering 11.5 and 7.1% of the genome, respectively, with 4,150 AS (5.2% of the genome) shared between the two cell lines (Fig. 5a, Supplementary Fig. 2a and Supplementary Table 1). The amplitude of the RFD shift (ΔRFD, see Methods section) across the segments ranged from 10 to 90%, indicating variable initiation efficiency (Supplementary Fig. 2b). As control, large (+)ΔRFD were detected around >90% of the early replication timing peaks previously identified in HeLa and GM06990 (see Methods section), confirming them as *bona fide* initiation zones (Fig. 2b,c, red arrows, Supplementary Fig. 3).

The large size of most AS was striking, with only a few tens <10 kb, suggesting that replication initiates mostly within broad zones and questioning whether site-specific origins exist in the human genome. First, we checked that the size estimate of AS was not biased by the presence of regions of lower read coverage at AS borders (Supplementary Fig. 4). Second, since HMM detection of AS required to compute RFD values within 15 kb sliding windows (Fig. 4), we checked whether this may overestimate the size of authentic site-specific origins. To begin with, we created in our profiles abrupt RFD shifts mimicking site-specific origins by deleting appropriate data points from AS. These were all detected by the HMM as 10–15 kb AS (Supplementary Fig. 5a,b). Next, we visually examined all detected AS<15 kb from native HeLa and GM06990 RFD profiles (Supplementary Fig. 6 and Supplementary Table 2). Among the 1,297 examined AS from HeLa cells, only 13 clearly showed an abrupt RFD shift spanning at most 1 kb and only 53 spanned from 1 to 5 kb. Many others showed a smooth 5–15 kb AS. However, a few hundreds may be interpreted either as broad (5 to 15 kb) initiation zones with ample signal dispersion or as tandems of site-specific origins. Similar results were obtained for GM06990 cells (Supplementary Fig. 6 and Supplementary Table 2). We also investigated the ability of the HMM to detect isolated site-specific origins of low efficiencies. Detection required a ΔRFD >20% (Supplementary Fig. 5c). Finally, we found that the extensive aneuploidy of the HeLa cancer cell line did not detectably affect the properties of initiation zones (Supplementary Fig. 7).

It has long been controversial whether replication initiates dispersively within broad zones or efficiently at narrowly localized sites in mammalian cells^1^. Two-dimensional (2D)-gel^30^,^31^ and bubble trap^4^ studies identified almost exclusively broad initiation zones, where each probed restriction fragment showed a composite Y (single fork) + bubble pattern, with a complete bubble arc thicker at its lower end and thinner at its upper end. This typical bubble signal was clearly different from that generated by a bacterial^30^ or a viral^31^ site-specific origin. In reconstruction experiments^30^, mixtures of bubbles generated from fixed origins located at different relative positions in the same fragment showed multiple, resolvable arcs even when origins were only 0.2–0.4 kb apart. Therefore, the continuity and thickness of mammalian bubble arcs could be only explained by the presence of bubbles generated at many positions (<1 kb apart from each other)^30^. This conclusion was independently confirmed by studying the migration of intentionally broken bubble fragments^31^, the size and origin of nascent strands from bubbles of increasing sizes^31^ and the co-electrophoresis of replication intermediates of the same plasmid replicated in bacteria and in *Xenopus* egg extracts^32^. The constant predominance of the Y signal implicated a low overall efficiency of initiation (∼1% per kb)^30^,^31^. In contrast, SNS studies detected narrow peaks of initiation along the genome, suggesting highly efficient, site-specific origins^6^,^7^,^8^,^9^,^10^,^12^. Even when multiple peaks could be clustered in zones^11^, most remained separated by longer distances than the typical SNS size (1.5–2.0 kb), suggesting distinct, highly preferred sites within as well as outside the zones. The genome-wide overlap between the zones mapped by bubble-trap or obtained by merging multiple SNS peaks remained modest, even using generous merging (<6 kb) and overlap (>1 nt) criteria^11^.

Using OK-seq, we clearly identified no more than a few tens of isolated, >20% efficient site-specific origins. In contrast, we identified thousands of broad (>15 kb) initiation zones. The quasi-linearity of the RFD shifts across most AS (Supplementary Fig. 2c) suggested that each active initiation zone supported a single but randomly located initiation event, reminiscent of the Chinese hamster *DHFR*^33^,^34^ and mouse *IgH*^35^ initiation zones. This interpretation is in good agreement with 2D-gel^34^, DNA combing^35^,^36^ and bubble-trap^4^ studies. The progressive shift in Okazaki fragment strandedness observed over tens of kb at most human AS is in strong contrast to the abrupt shifts reported at *S. cerevisiae* origins^27^,^28^, which are defined by short specific sequences.

We caution that we would be unable to detect weak (ΔRFD<20%) but site-specific origins, and that closely spaced tandems of site-specific origins would be detected as broad AS. If tandems of site-specific origins detected by SNS analysis were to account for most AS, however, we should detect a strong enrichment of SNS inside AS. As shown in a later section, bubble-trap origins were clearly more homogeneously enriched than SNS within AS. We, therefore, favour the alternative hypothesis that AS contain multiple inefficient sites spaced at <1 kb intervals. The average slope of AS suggests an overall initiation rate of ∼1% per kb (Supplementary Fig. 2b), consistent with 2D-gel studies. Although precise measurements of initiation rate fluctuations across individual AS will require further work, we expect that the dispersive nature of initiation observed across AS and bubbles would escape detection by SNS peak-searching algorithms. The lower overlap of SNS than bubbles with AS is further examined later in this manuscript, after characterization of AS location with respect to active genes.

### Many replication initiation zones flank active genes

AS were predominantly intergenic and their ends bordered expressed genes more often than expected by chance (Fig. 5a-c); in HeLa cells, 19% of AS coincided (> 50%, 80% on average) with non-transcribed regions separating two active genes (type 1 AS), 27% were flanked (<20 kb) by one active gene (type 2 AS) and 35% were not flanked (<20 kb) by active genes (type 3 AS); many of the remaining 20% (type 4 AS) corresponded to type 1 or 2 AS with presumably mis-annotated transcripts (Fig. 5d, Supplementary Fig. 8). Similar results were observed for GM06990 cells (Supplementary Fig. 9d,e). Shared AS between the two cell lines were enriched in type 1 whereas cell-type-specific AS were enriched in type 3 (Supplementary Table 1). No particular positioning of inactive genes was detected for any AS type (Supplementary Fig. 10). Types 1 and 2 replicated almost exclusively in early S phase whereas type 3 replicated predominantly late (Supplementary Fig. 9d,e). Isolated genes expressed in only one cell type were flanked by AS only in that cell type (Fig. 5e,f). Initiation was routinely (70–90%) detected between active genes separated by >20 kb, but less often within smaller intergenes; a preference for divergent over tandem over convergent gene pairs was observed, particularly in small intergenes (Fig. 5g). This preferential initiation upstream of active genes contributed to a significant co-orientation of replication with transcription (Fig. 5h), extending previous findings in skew N-domains^19^. These data show that many dispersive initiation zones consist of large DNA segments precisely circumscribed by active genes. They are in agreement with early studies of a few model loci^1^,^33^,^34^,^37^ and with the recent key observation that transcription shapes a dramatic redistribution of the MCM2–7 complex, a core component of the replicative helicase marking potential origin sites, to exclude MCM2–7 from transcribed genes before S-phase entry in *D. melanogaster*^38^. However, this is probably not the single mechanism for specifying initiation zones. Using available global run-on sequencing (GRO-seq) and cap analysis of gene expression (CAGE) data^39^, we detected only a small amount of non-coding transcripts in the type 2 AS flanks that are devoid of active genes, and their level was not markedly higher than inside the AS; furthermore, we did not detect any significant level of non-coding transcripts in the non-genic flanks of type 3 AS (Supplementary Fig. 11). Therefore, initiation zones borders can also be delimited by non-transcriptional mechanisms.

### Many replication termination zones overlap active genes

The HMM also detected 9,440 (HeLa) and 5,715 (GM06990) DS (8–2600, kb) covering 49.5% and 39.4% of the genome, respectively, with 5,000 (35% of the genome) shared between the two cell lines (Supplementary Figs 2a and 9f,g and Supplementary Table 1). In early S phase, DS predominantly overlapped transcribed genes, as expected from initiation predominance in intergenes; in late S phase, DS predominantly consisted of large non-expressed DNA regions (Fig. 5a,i, Supplementary Figs 1 and 9f,g). Many DS were directly contiguous to AS, implying that termination often occurs anywhere between adjacent AS.

### Evidence for large unidirectional replication zones

The HMM detected 745 (HeLa) and 1,101 (GM06990) FS (20–1,200 kb). Most of these (617 and 849, covering 4.1% and 7.3% of the genome, respectively, 235 shared segments) showed a strongly (>80%) favoured replication direction (|RFD|>0.6; Supplementary Fig. 2d; for an example see region 68.9–69.2 Mb of HeLa profile in Fig. 5f). These high-|RFD| FS bordered initiation zones, often started at an active transcription start site (TSS), and were enriched in expressed genes co-oriented with replication (Fig. 5j,k). These segments are likely devoid of origins and termini and are almost always replicated from their upstream initiation zone. They separate AS from DS so that the forks that traverse them terminate away, rather than at any distance, from their upstream initiation zone.

### Most initiation sites are not associated with G4 or CGIs

**Figure 6:**
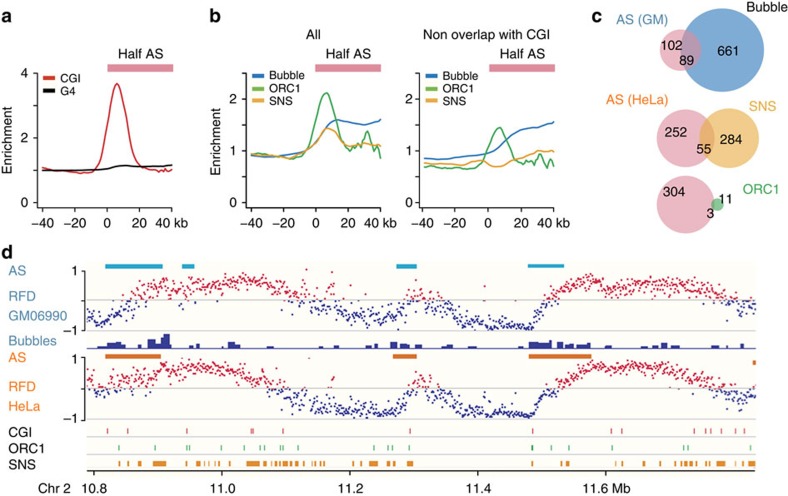
Comparison of initiation zones with previous origin mapping data. (**a**) Enrichment ratios (see Methods section) of CGIs (red) and G4s (black) around HeLa AS borders; pink box indicates position of half AS. (**b**) Enrichment ratios of bubbles^4^ (GM06990, blue), ORC1 (ref. 13) (HeLa, green) and SNS^11^ (HeLa, yellow) peaks around AS borders of the same cell type, for all peaks (left) or for peaks not overlapping CGIs (right). (**c**) Overlap (in Mb) of AS with bubbles, SNS and ORC1 peaks. (**d**) Exemplary profiles showing distribution of bubbles, CGIs, ORC1 and SNS peaks with respect to AS (GM06990, teal blue lines; HeLa, orange lines).

Previous studies^4^,^6^,^7^,^8^,^9^,^10^,^11^,^13^ have reported an association of mammalian origins with TSSs and associated elements such as CpG islands (CGIs) and G-quadruplex (G4) elements. The degree of enrichment was variable, and concerns that it may arise from the failure of λ-exonuclease to eliminate G-rich sequences in SNS-contaminating background DNA were raised^1^,^3^,^40^. We found that CGIs were enriched at the borders of AS, especially AS shared by HeLa and GM06990, but not inside AS (Fig. 6a, Table 1). This reflected the frequent occurrence of CGIs around housekeeping gene TSSs. There was only a weak, if any, enrichment of G4s inside AS (Fig. 6a). Therefore, most initiation sites within initiation zones are not associated with CGIs or G4s.

### Initiation zones better align to bubbles than to SNS and ORC

Two remarkable characteristics of AS were their sharp boundaries and their linearity, suggesting a fairly homogeneous internal rate of initiation falling off markedly at the borders. On average, short nascent strands (SNSs)^9^,^11^, ORC1-binding sites^13^ and trapped bubbles^4^ were ∼2 times more abundant within than outside AS from the same cell type (GM06990 for bubbles; HeLa for SNSs and ORC1 peaks; Fig. 6b–d), consistent with a higher efficiency of initiation within AS and a predominance of termination within DS. However, SNSs were not uniformly enriched along AS but peaked at their borders; these peaks disappeared after masking CGIs (Fig. 6b). ORC1-binding sites were also enriched at the borders but independently of CGIs (Fig. 6b) and of AS size (Supplementary Fig. 12). Similar results were obtained when TSSs from promoter regions that are both positive for ORC1 and enriched in SNS were analysed; the bimodal SNS enrichment pattern previously observed^41^ on both sides of such TSS/ORC1 sites was due to association of SNS with CGIs and not with ORC1 (Supplementary Fig. 13). Both 5′ and (to a lesser extent) 3′ gene ends contributed to ORC1 enrichment at AS borders (Supplementary Fig. 14). Globally, the density of ORC1 peaks was 2.4-fold higher in AS than in DS and FS. When the density of ORC1-binding sites was plotted along the segments delimited by consecutive AS, ORC1 enrichment peaked at the AS and declined in a distance-dependent manner in later-replicating sequences (Supplementary Fig. 15), consistent with the reported correlation of ORC1 density with replication timing^13^. Finally, trapped bubbles were enriched throughout the AS independently of CGIs (Fig. 6b,c), at all stages of S phase (Supplementary Fig. 16) and for all AS types (Supplementary Fig. 17).

The abundance of SNSs at CGIs^6^,^9^,^11^ suggested a high efficiency of initiation at these sites but this was neither reflected in bubble abundance nor by upshifts in RFD profiles. Only the trapped bubbles, and to a lesser extent the CGI-negative SNS, showed the homogeneous enrichment inside AS predicted by the linear slope of AS. Our data are consistent with the proposal that enrichment of SNS in CGI and other CG-rich sequences is due to an intrinsic bias of the λ-exonuclease technique^1^,^3^,^40^. Removing the CGI-associated SNS improved the homogeneity of SNS enrichment throughout AS but CGI-negative SNS were much less enriched than bubbles (Fig. 6b). The possibility that SNS peak detection algorithms overlooked a broad but uniform enrichment of SNS within AS was excluded by analysis of SNS raw data, which did not improve the enrichment inside AS (Supplementary Fig. 18). We, therefore, favour the hypothesis that bubbles are a more representative sample of human replication initiation sites than SNS. Together, the bubble-trap and OK-seq data favour the view that replication usually initiates within broad zones of multiple inefficient sites rather than at highly efficient specific sites.

The ORC1 signal was concentrated at AS borders rather than spread throughout AS as expected, if initiation events were to coincide strictly with ORC-binding sites. First, ORC has other nuclear functions than replication initiation and some ORC-binding sites may not be origins. Second, metazoan ORC binds DNA *in vitro* with no sequence specificity^42^,^43^,^44^ and the chromatin immunoprecipitation-sequencing (ChIP-seq) enrichment of ORC at specific chromatin sites is quite moderate in comparison to other chromatin-binding proteins^45^. Indeed, the number of reads per ORC1 peak is relatively low in Dellino *et al*.^13^, which is the only reported genome-wide study of human ORC-binding sites. Clusters of ORC-binding sites might be difficult to detect if enrichment is too low compared with non-origin regions.

In addition, the modest overlap observed between AS- and ORC-binding sites could result from initiation away from ORC. Multiple observations suggest that human MCM2–7 are broadly distributed throughout the nucleus and exhibit little colocalization with ORC^46^,^47^ and that initiation events can occur at MCM2–7 complexes spread over a broad zone around ORC^48^,^49^,^50^,^51^ in metazoan cells. Since MCM2–7 interact with only one side of ORC during loading^52^, ORC bound at gene ends may load multiple copies of the MCM2–7 complex only on its non-genic side. Alternatively, MCM2–7 may be dispersively loaded on both sides of ORC^48^,^49^,^50^, but erased from transcribed regions in G1 phase^37^,^38^,^53^,^54^, ultimately leading to the formation of broad intergenic initiation zones. In strong support of this model, MCM2–7 loaded in late G1 in *Drosophila* cells show a bimodal distribution of enrichment along transcribed and non-transcribed sequences, due to their specific displacement from transcribed genes and their unability to be re-established or to translocate into transcribed genes after the G1/S transition^38^. This model, therefore, explains why the overlap of ORC1 with initiation sites is only partial and why many AS abut transcribed genes.

### Initiation zones are enriched in specific chromatin marks

**Figure 7:**
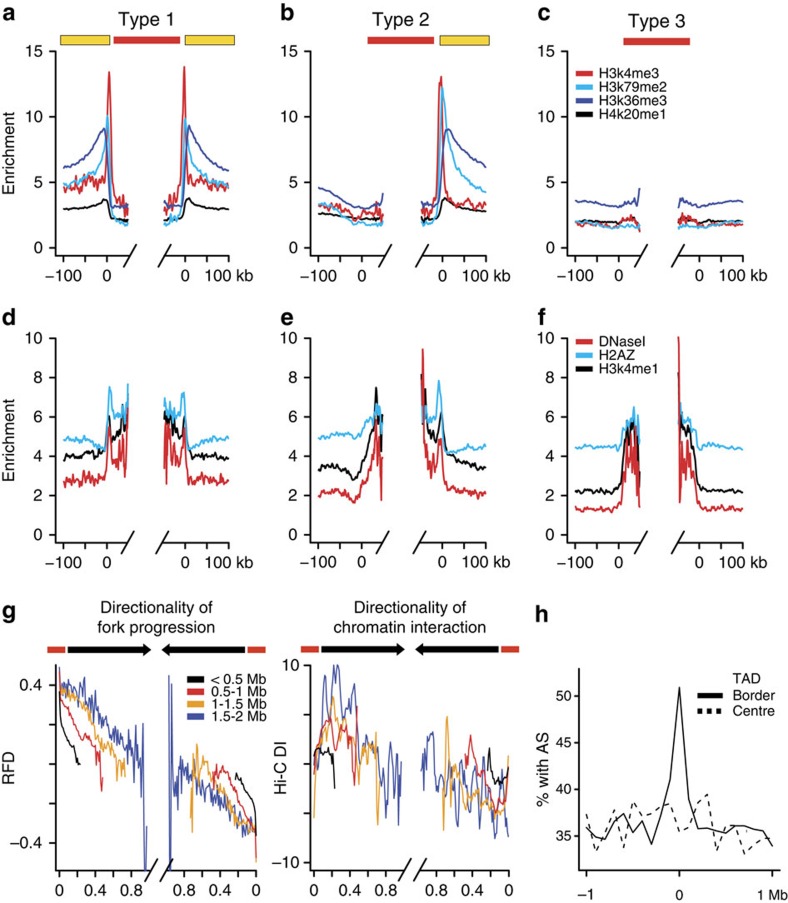
Chromatin structure impact on replication landscape. (**a**-**f**) Densities of signal enrichment of indicated histone marks or DNaseI HSSs around HeLa Type 1 (**a**,**d**), Type 2 (**b**,**e**) and Type 3 (**c**,**f**) AS; yellow boxes, active genes flanking AS (red lines). (**g**) Mean HeLa RFD (left) and Hi-C directionality index (DI, see Methods section, right) between consecutive AS separated by indicated distances. Black arrows indicate the co-orientation of both directionalities between AS (red). (**h**) Percentage of genomic segments (100 kb bin) overlap with HeLa AS around the borders (line) or centres (dash) of TADs shared by IMR90 and H1ESC^61^.

As expected from the bordering of many AS by expressed genes, epigenetic marks associated with active TSSs (H3K4me2 and 3, H3K9ac, H3K27ac) and gene bodies (H3K79me2 and H3K36me2) were enriched at the boundaries and flanks of AS, respectively, but not inside them (Fig. 7a–c and Supplementary Fig. 19 for HeLa, Supplementary Fig. 20 for GM06990). H4K20me1, previously associated with SNSs^11^, was only weakly enriched at active TSSs and not inside AS (Fig. 7a–c). In contrast, DNAse I hypersensitive sites (HSSs), H3K4me1 and histone variant H2A.Z were more abundant within than outside AS, irrespective of the presence or absence of flanking transcribed genes (Fig. 7d–f for HeLa, Supplementary Fig. 21a–c for GM06990). Using available GRO-Seq and CAGE data^39^, we did not detect non-coding transcripts flanking type 3 AS and very little such transcripts in the type 2 AS flanks that are devoid of active genes (Supplementary Fig. 11). We neither detected a depletion of nucleosomes within AS (Supplementary Fig. 21d–f). Therefore, the DNAse I HSSs-H3K4me1-H2A.Z trio is a more universal characteristic of initiation zones than the presence of flanking transcribed sequences. These three marks often co-localized when individual loci were examined (Supplementary Fig. 21g). Interestingly, H3K4me1 is present mostly at poised or active enhancers^55^ and H2A.Z, a chromatin component at DNAse I HSSs^56^ has been proposed as an important regulator of enhancer activity^57^. The correlation of DNAse I HSSs with initiation zones is in agreement with a model suggesting that this mark is the best predictor of replication timing, albeit at low resolution^58^. At present, however, such predicted replication profiles^58^ are not precise enough to foretell initiation zones (Supplementary Fig. 1b).

### TAD borders are enriched in initiation zones

It has been recently reported^59^ that the boundaries of cell-type-specific replication timing domains often coincide with the boundaries of insulated compartments of chromatin interaction, identified by high-resolution chromatin conformation capture (Hi-C)^60^, and dubbed ‘topologically associated domains' (TADs)^61^. When the directionality of chromatin interactions^61^ and RFD were mapped between the midpoints of consecutive AS, both exhibited similar N-shaped profiles with opposite extrema at domain borders and null values at centres (Fig. 7g for HeLa, Supplementary Fig. 22a,b for GM06990). Remarkably, the borders but not the centres of TADs were strongly associated with AS (Fig. 7h for HeLa, Supplementary Fig. 22c for GM06990). These findings imply that replication of TADs often starts at their borders and propagates to their center.

### Initiation events outside AS

**Figure 8:**
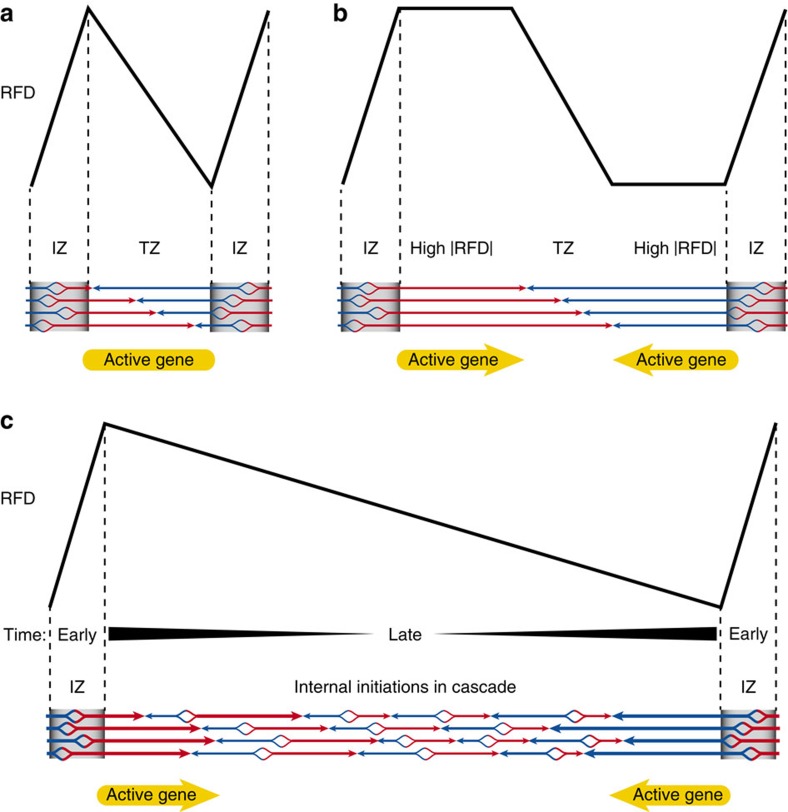
Model for replication of N-shaped RFD domains. (**a**) When initiation zones are close enough from each other such as in gene-rich early-replicating regions, the temporal and spatial stochasticity of origin firing may cause emanating forks to converge at different positions spanning the entire intervening segment. This results in a straight N-shaped RFD profile. (**b**) Flat segments of high |RFD| are generated when initiation zones are distant and efficient enough to prevent the fork emanating from one initiation zone to reach the next one before it can fire, resulting in a ‘crenellated' N-shaped RFD profile. This model implies that high |RFD| FS are devoid of origins. Such segments are enriched in active genes co-oriented with replication fork progression. (**c**) Large, straight N-shaped RFD profiles can form between very distant initiation zones if cascades of intervening background origin firing initiate at their borders and propagate to their center at an increasing rate during S phase. This results in a progressive inversion of RFD along the segment^15^ (see text).

The number of detected AS was significantly lower than all current estimates of the number of initiation events per haploid genome^4^,^9^. Thus, initiation likely occurs outside AS but without creating visible RFD upshifts. We, therefore, propose that the human genome presents a superposition of specific, ‘master' initiation zones (AS) and a background of more dispersed, less efficient origins (Fig. 8), in agreement with the widespread occurrence of inefficient origins detected by other methods outside AS (Fig. 6c). Thus, the predominance of termination over initiation within DS, which arises from the net inward flux of forks emanating from adjacent AS, is only relative. In small DS, background initiation may be negligible, and the linear inversion of RFD across DS would simply reflect the population average of forks emanating from adjacent AS and terminating at different sites (Fig. 8a,b). In longer DS, where background initiation becomes significant (Fig. 8c), the linear inversion of RFD can be quantitatively explained by a ‘cascade' model for sequential activation of internal origins with increasing synchrony from domain borders to center^15^,^25^,^26^. In this model, background origins close to domain borders fire in a sequential wave from borders to center, so that center-oriented forks travel longer distances than border-oriented forks before their merging (high |RFD|). Closer to domain centres, background origins fire later and more synchronously so that forks moving in both directions travel more similar distances (low |RFD|). The master initiation zones are often adjacent to, but excluded from, transcribed sequences and are associated with open chromatin marks even when they are not flanked by active genes. The N-shaped pattern of chromatin interaction directionality observed between consecutive master origins at TAD borders may underlie the sequential activation of intervening background origins resulting in N-shaped RFD profiles.

A recent genome-wide, single-molecule analysis of chromosomal DNA replication in *S. pombe*^62^ concluded that early origins are focused but later origins are more dispersive, that replication propagates from the earliest origins by firing additional origins in adjacent regions and that the rate of origin firing strongly increases as S phase progresses. These results strikingly converge with the cascade model proposed for origin activation in the human genome^15^,^25^,^26^ based on previous work in human cells and other eukaryotes^48^,^63^,^64^,^65^. In *S. pombe*, however, the earliest initiation events do not always appear at the same locations on the chromosomes, in contrast to the ‘master' initiation zones observed at the borders of N/U-domains and in this work.

In summary, the novel RFD maps produced here confirm and extend previous views of chromosomal domain organization inferred from replication timing, nucleotide compositional skew and chromatin conformation data. They provide new insight into replication initiation and termination zones genome-wide and help to clarify controversies between previous origin-mapping studies. Replication initiation and termination sites have been mapped previously by sequencing Okazaki fragments in *S. cerevisiae*, but this necessitated the introduction of conditional lethal mutations in ligase and checkpoint genes to massively increase Okazaki fragment abundance^27^,^28^. The methodology presented here, which does not require ligase inactivation or any other cell modification, can be applied to a wide variety of organisms and cell types to study how replication patterns change during differentiation, tumorigenesis and evolution.

## Methods

### EdU labelling

Asynchronously growing 2–3 × 10^8^ HeLa (MRL2 strain, a gift from O. Bensaude, IBENS) or 10^9^ GM06990 cells (Corriell) were labelled by adding 20 μM EdU (Jenabioscience) to growth media for 2 min. Adherent HeLa cells were washed with ice-cold 1 × phosphate-buffered saline (PBS) and harvested by scrubbing with a cell lifter. Non-adherent GM06990 cell suspensions were chilled in ice-cold water bath for 10 min and collected by centrifugation at 400 *g* for 10 min at 4 °C. As an alternative to EdU, 5-ethynyl-2′-deoxycytidine (EdC) was also used with identical results.

### Genomic DNA preparation

Genomic DNA was extracted by standard proteinase K/phenol extraction followed by ethanol precipitation^66^, dissolved in 4 ml of 10 mM Tris-HCl, pH 8.0, 1 mM EDTA and stored at 4 °C.

### Okazaki fragment isolation and biotinylation

Genomic DNA was denatured at 94 °C for 10 min, chilled on ice and size-fractionated on 5–30% (w/v) linear sucrose gradients in 10 mM Tris-HCl pH 8.0, 1 mM EDTA, 100mM NaCl in a Beckman Ultracentrifuge SW28 rotor for 17 h at 26,000 *g* at 20 °C (ref. 67). Fractions containing <200 b-strands (typically upper 8 ml) were concentrated on Amicon Ultra 10k centrifuging filter (Millipore), buffer exchanged twice with 5 ml of water and concentrated to 390 μl. Click reaction was performed in the dark in a final volume of 500 μl by sequential addition of 10 mM Tris-HCl pH 8.0, 2 mM CuSO4, 1 mM biotin-TEG-Azide, 10 mM sodium ascorbate, incubation for 45 min at room temperature, followed by precipitation with ethanol and resuspension in 90 μl water. RNA was hydrolysed for 30 min at 37 °C by adding 10 μl of 2.5 M NaOH, followed by neutralization with 10 μl of 2.5 M acetic acid and purification on Micro Bio-Spin Columns with Bio-Gel P-30 (Bio-Rad). DNA 5′ ends were phosphorylated with 30 U of T4 Polynucleotide kinase (Fermentas) for 30 min at 37 °C, followed by enzyme inactivation for 10 min at 75 °C, ethanol precipitation and resuspension in 20 μl of water.

### Sequencing adaptor ligation and Okazaki fragment capture

Double-stranded adaptors with single-stranded random hexanucleotide overhangs were generated by annealing equimolar amounts of complementary oligonucleotides in 50 mM NaCl in a thermal cycler by heating at 95 °C for 5 s and cooling down to 4 °C at 0.1 °C s^−1^. The sequences of oligonucleotides pairs were as follows:

A1_watson_: 5′-ACACTCTTTCCCTACACGACGCTCTTCC-3′;

A1_crick_ : 5′-NNNNNNGGAAGAGCGTCGTGTAGGGAAAGAGTG-3′;

A2_watson_ : 5′ [Phos]-AGATCGGAAGAGCACACGTCTGAACTCCAGTCA[ddC]-3′;

A2_crick_: 5′-TGACTGGAGTTCAGACGTGTGCTCTTCCGATCTNNNNNN[ddC]-3′.

The A1_watson_ and A2_watson_ sequences were identical to the two strands of the Illumina TruSeq adaptor except that A1 was truncated by five bases to reduce unwanted annealing with A2 during library preparation^68^.

5′-phosphorylated single DNA strands were annealed to 80 pM of each adaptor in a thermal cycler by heating at 65 °C for 10 min and cooling to 16 °C for 5 min. Ligation was performed at 16 °C overnight in 40 μl of ligase buffer added with 500 mM betaine, 5% PEG8000 and four Weiss Units of T4 DNA Ligase (Fermentas). Biotinylated fragments were captured with 200 μg of Dynabeads MyOne Streptavidin T1 according to the manufacturer's protocol (Life BioSciences). Unligated adaptors were removed by washing five times with 5 mM Tris-HCl pH 7.5, 0.5 mM EDTA, 1 M NaCl, 0.05% Tween 20, twice with 10 mM Tris-HCl pH 8.0, 1 mM EDTA, 0.05% Tween 20 and once with water. Adaptor annealing and ligation were then repeated using 20 μl of bead suspension and 40 pM of each adaptor. Beads were washed as above and resuspended in 20 μl of 10 mM Tris-HCl pH 8.0.

### Library amplification and sequencing

Libraries were amplified in 12 cycles of PCR using 10 μl of bead suspension as a template, Phusion polymerase (NEB) and the following primers. The forward primer was PE PCR Primer 1.0 truncated by 5 bases:

5′-ATGATACGGCGACCACCGAGATCTACACTCTT TCCCTACACGACGCTCTTCC-3′. The reverse primer was TruSeq Index primer:

5′-CAAGCAGAAGACGGCATACGAGAT-3′******5′-GTGACTGGAGTTCAGACGTGTGCTCTTCCGATCT-3′ (* indicates Illumina index). PCR products were purified on Qiagen MinElute columns and fragments from 150 to 400 bp were size-selected from 4% small fragment agarose gel (Eurogentec) using Qiagen gel extraction kit. Generation of clusters was performed on Illumina cBot following standard protocols except that the sequencing primer was replaced by 5′-ACACTCTTTCCCTACACGACGCTCTTCC-3′ (ref. 68). Sequencing was performed on Hiseq 1000 sequencer (single read 51 pb) according to standard procedures.

### Sequencing data

All Illumina sequencing runs were performed at IMAGIF high-throughput sequencing platform. Sequence reads were identified and demultiplexed using the standard Illumina software suite and adaptor sequences were removed by Cutadapt (version 1.2.1). Reads >10 nt were aligned to the human reference genome (hg19) using the BWA (version 0.7.5a) software with default parameters. We considered only uniquely mapping reads and removed PCR duplicate reads using Picard (http://picard.sourceforge.net).

### Replication fork directionality

RFD was computed for each 1 kb window as follows:


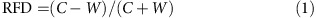


where *C* and *W* correspond to the number of reads mapped on Crick and Watson strand, that reveal, respectively, the proportions of rightward- and leftward- moving forks within each window. Since the total amount of replication on both strands should be constant across the genome, we normalized the difference between two strands by the total read count to account for variation in read depth due to copy number, sequence bias and so on. RFD ranges from −1 (100% leftward moving forks) to +1 (100% rightward moving forks) and 0 means equal proportions of leftward- and rightward-moving forks. Replicate experiments produced RFD that strongly correlated to each other (Supplementary Fig. 1; for HeLa cells, *R*=0.92 (Pearson), *P*<10^−15^ (*t*-test) and for GM06990 cells, *R*=0.93, *P*<10^−15^). Similar correlations were observed between EdU and EdC profiles.

### Segmentation of RFD profiles

A four-state HMM was used to detect within the RFD profiles the AS, DS and FS segments representing regions of predominant initiation (‘Up' state), predominant termination (‘Down' state) and constant RFD (‘Flat1' and ‘Flat2' states; see Supplementary Fig. 9a for a graphic representation). The RFD values were computed within 15 kb sliding windows (stepped by 1 kb across the autosomes). The HMM used the ΔRFD values between adjacent windows (that is, ΔRFD_*n*_=(RFD_*(n+1)*_−RFD_*(n)*_)/2 for window *n*). Windows with <30 reads on one strand were masked. The ΔRFD values were divided into five quantiles and the HMM package of R (http://www.r-project.org/) was used to perform the HMM prediction with probabilities of transition and emission indicated in the Supplementary Fig. 9b,c. Only segments reproducibly identified in both biological replicates were retained. The choice of a 15kb sliding window is based on a compromise between spatial resolution and reproducibility of AS detection among biological replicates (Fig. 4). The efficiency of AS was estimated as:


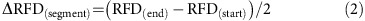


where RFD_(start)_ and RFD_(end)_ correspond, respectively, to the RFD values computed in 5 kb windows around left and right extremities of each segment.

### Replication timing profiles

The raw sequencing data and the peaks identified as the local maxima in the wavelet-smoothed signal profiles of HeLa and GM06990 cells were downloaded from the UW_Repli-Seq track at the UCSC genome browser (http://genome.ucsc.edu/ENCODE/). When necessary, genomic coordinates were remapped to hg19 using LiftOver. Replication timing was computed in 100 kb sliding windows (stepped by 1 kb) along the human genome as described^21^. The enrichment of sequence reads along the genome was computed in each S phase compartment and the ratio *S*_50_, defined as the fraction of the S phase (0<*S*_50_<1) at which 50% of DNA is replicated in a defined region, was computed by linear interpolation of enrichment values in the six S phase compartments. The mean replication time of each HMM-detected AS, DS and FS, was assigned to one of the following four periods of S phase: S1 (0.1<*S*_50_≤0.3), S2 (0.3<*S*_50_≤0.5), S3 (0.5<*S*_50_≤0.7) and S4 (0.7<*S*_50_≤0.9). The coordinates of replication timing U-domains were retrieved from ref. 25.

### Nucleotide compositional skew profile

The nucleotide compositional skew (GC and TA skews defined as *S*_GC_=(*G−C*)/(*G+C*), *S*_TA_=(*T−A*)/(*T+A*) and total skew *S*=*S*_GC_+*S*_TA_) was computed in 1 kb adjacent windows with the repeat masked sequences as described before^19^,^24^ and the coordinates of skew N-domains were retrieved from ref. 19.

### Comparison with transcription data

Annotations of the human genome (version 73) were retrieved from the Ensembl Genome Brower (http://www.ensembl.org). The gene transcription level of each cell type was obtained from the Expression Atlas (www.ebi.ac.uk/gxa), which uses the transcription data generated by the ENCODE project to compute the average FPKM (fragments per kilobase per million reads) using biological replicates. The raw RNA-sequencing data (PolyA+, Long PolyA− and short Poly A−) were downloaded from the UCSC genome browser. The GRO-Seq and CAGE data of HeLa cells were retrieved from ref. 39. Genes entirely included within another gene in the same orientation were removed. To reveal the relation between replication initiation and gene transcription, the distance between each initiation zone extremity to the closest active or inactive gene extremities was computed for each cell type. Random simulations were performed to evaluate the significance of the results, taking into account the heterogeneity of AS and gene distributions with respect to replication timing. First, for each chromosome, the genome was segmented into regions according to replication timing, assigned as S1, S2, S3 or S4. All regions presenting the same Si were then connected, leading to four virtual chromosomes. For each virtual chromosome, the AS and regions separating adjacent AS (IAS) were shuffled, alternating AS and IAS. The number, size distribution and replication timing (S1, S2, S3 and S4) of AS and IAS were thus conserved for each replication timing compartment. The distance between each shuffled AS extremity and the closest active or inactive gene extremities was then computed. 1,000 simulations were performed to obtain the mean values of this neutral model. Genes specifically expressed in HeLa or GM06990 and without another active gene located <50 kb upstream (or downstream) of TSS (or TTS) were used in Fig. 5e to study the association of cell-type-specific RFD profiles with cell-type-specific transcription.

### Sequence features and replication origin mapping data

The coordinates of CGIs were downloaded from UCSC genome browser and the positions of potential G4s were detected as described in ref. 11, specifying the length of the spacer between four tracks of GGG or CCC with spacer of size 2–4 nt. Published HeLa SNS-seq^11^, HeLa ORC1 ChIP-seq^13^ and GM06990 bubbles^4^ data (detected peaks) were used to compute enrichments along AS and surrounding regions in adjacent 1 kb windows. To compare the enrichments, the density profiles were normalized by the average density within the examined region (Fig. 6a,b, Supplementary Figs 12–16). In Supplementary Fig. 13a–c, raw HeLa SNS-seq^9^ and ORC1 ChIP-seq^13^ data were used to compute enrichments around TSS in adjacent 100 nt windows.

### Histone and open chromatin marks

The data of signal enrichment for histone marks, including H2A.Z, H3K4me1, H3K4me2, H3K4me3, H3K9ac, H3K9me3, H3K27ac, H3K27me3, H3K36me3, H3K79me2 and H4K20me1 for HeLa and GM12878 (closely related to GM06990) cells were downloaded from the Board Histone track at the UCSC genome browser. The data for nucleosome positioning (MNase-seq) in GM12878 cells were downloaded from the Stanf Nucleosome track at the UCSC genome browser. The DNase I-seq data (UW DNase I HS data track) and the DNase I hypersensitivity uniform peaks of HeLa and GM06990 cells were retrieved from ENCODE.

### Hi-C chromatin interaction data

The Hi-C data of HeLa^69^ and GM06990 (ref. 60) cells were retrieved and the Directionality Index (DI) was computed as described^61^:


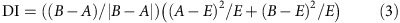


where *A* and *B* are the number of reads that map from a given 40 kb bin to the upstream and downstream 2 Mb, respectively, and *E*, the expected number of reads under the null hypothesis (*E*=(*A*+*B*)/2). The coordinates of the TADs of H1 human embryonic stem cells and IMR90 fibroblasts were retrieved from ref. 61.

## Additional information

**Accession codes:** OK-Seq data have been deposited at NCBI Sequence Read Archive under accession no: SRP065949. We have developed a web-based database to visualize the high-resolution RFD profiles that is available at http://157.136.54.88/cgi-bin/gbrowse/gbrowse/okazaki_ref/.

**How to cite this article**: Petryk, N. *et al*. Replication landscape of the human genome. *Nat. Commun.* 7:10208 doi: 10.1038/ncomms10208 (2016).

## Supplementary Material

Supplementary Information Supplementary Figures 1-22, Supplementary Tables 1-2 and Supplementary References

## Figures and Tables

**Figure 1:**
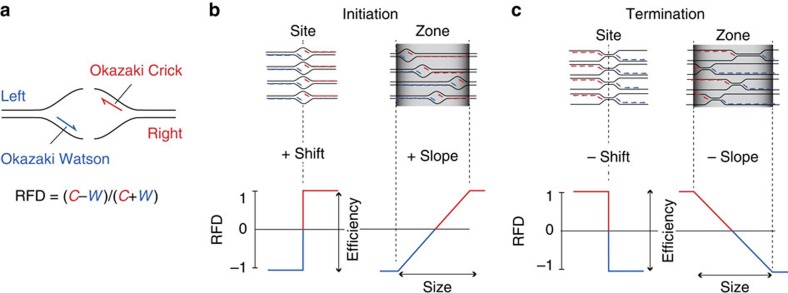
Principle of RFD mapping by OK-Seq. (**a**) Schematic leftward (blue) and rightward (red) forks with Okazaki fragments. RFD is computed as the difference between the proportions of rightward- and leftward-moving forks in 1 kb windows. (**b**) Expected RFD profiles for a fixed origin or a broad initiation zone. (**c**) Expected RFD profiles for a fixed terminus or a broad termination zone. The bubbles in **a**,**b** and X-shaped structures in **c** indicate the location of potential initiation and termination sites. The red and blue lines inside these structures indicate the Okazaki fragments synthesized during the progression of each fork. These Okazaki fragment populations are randomly sampled when extracted from asynchronously growing cell populations.

**Table 1:** Number of common and cell-type-specific HeLa and GM06990 AS overlapping CGIs or not.

	**Common**	**HeLa specific**	**GM06990 specific**
All	4,186 (4,111)	5,650	1,573
CGI−	1,710 (1,707)	3,416	1,039
CGI+	2,476 (2,404)	2,234	534
CGI%	59%	40%	34%

AS, ascending segment; CGI, CpG island.
