# Supplementary material for: Replication landscape of the human genome
Source: Nat Commun. 2016 Jan 11;7:10208. doi: 10.1038/ncomms10208 (PMC4729899; doi:10.1038/ncomms10208)
Supplement: Supplementary Information — Supplementary Figures 1-22, Supplementary Tables 1-2 and Supplementary References [file ncomms10208-s1.pdf]

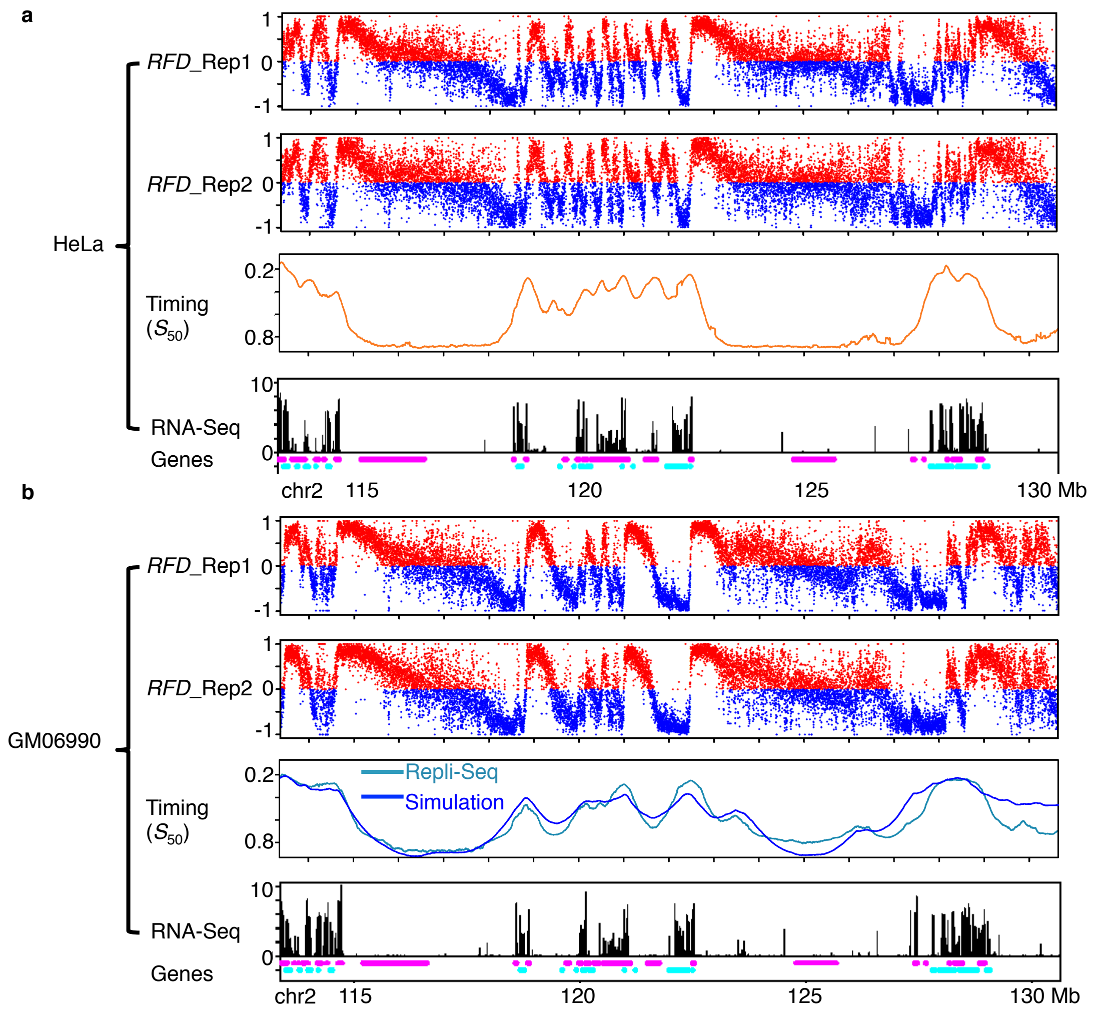

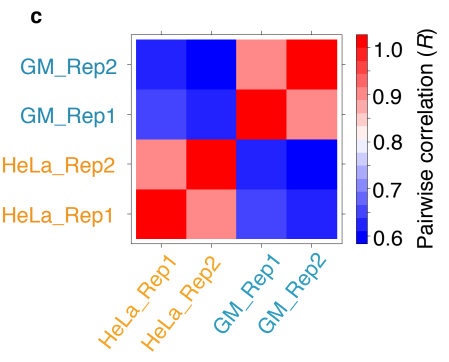

d

Sample	Sequencing device	Single/ Paired ends	Total reads	Unique mapped reads
HeLa_Rep1	HiSeq	SE	1,701,698,286	1,255,293,705
HeLa_Rep2	HiSeq	SE	1,683,709,895	1,241,704,542
GM06990_Rep1	HiSeq	SE	1,488,904,972	1,200,009,561
GM06990_Rep2	HiSeq	SE	1,382,367,423	868,939,160

Supplementary Figure 1. Reproducibility of *RFD* profiles and comparison with replication timing and gene expression. Two experimental replicate *RFD* profiles from the same region as in Figure 2b,c (Rep1 and Rep2), performed (**a**) with HeLa cells and (**b)** with GM06990 cells. For each cell type the Rep1 and Rep2 profiles were almost identical; the Pearson correlation coefficients were 0.92 for HeLa and 0.93 for GM06990 (**c**). S_{50}, replication timing profile computed as before[1] with Encode Repli-Seq data[2]. The dark blue line superimposed on the GM S_{50} profile is a previous simulation[3] based on DNAse I HSS distribution. RNA-Seq data were retrieved from (Caltech RNA-Seq, ENCODE[4]). Genes transcribed rightward (leftward) are indicated as magenta (cyan) lines. (**d**) Number of sequencing reads for HeLa and GM06690 replicates.

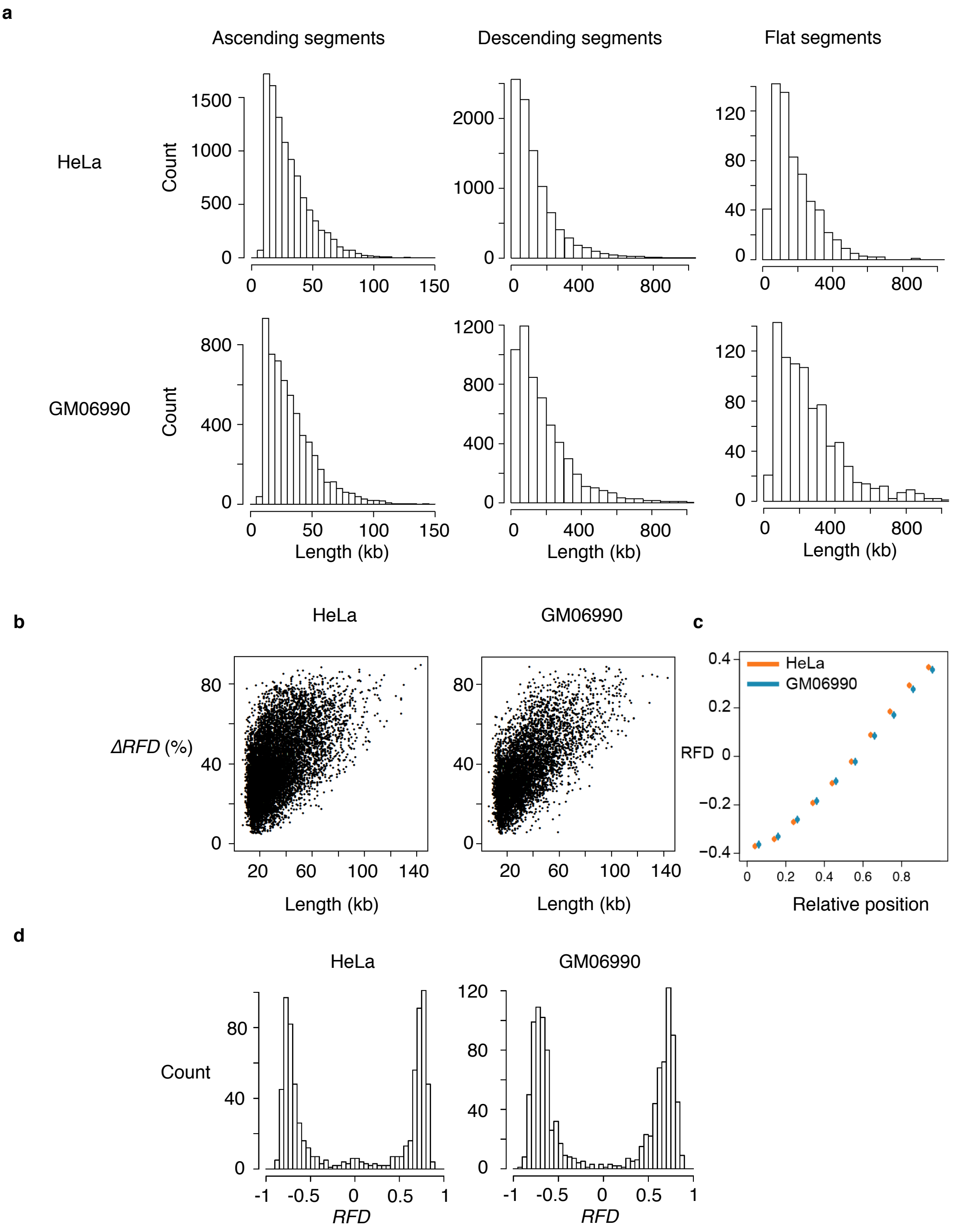

Supplementary Figure 2. Properties of ascending, descending and flat segments (AS, DS, FS) of HeLa and GM06990 *RFD* profiles. **a**, length distributions. **b**, distributions of *RFD* shift (initiation efficiency), $\Delta RFD = (RFD_{end} - RFD_{start}) / 2$ (Methods), of individual AS according to AS length. Efficiency is broadly proportional to length. The mean local efficiency is ~0.01 kb^{-1}, quite comparable to the DHFR[5] and IgH[6] initiation zones. **c**, mean *RFD* profile of rescaled AS from the indicated cell type. Standard errors of mean (*SEM*, bars) are very small (<0.005), showing the quasi-linearity of both AS profiles. The mean AS firing efficiency (ΔRFD) is ~0.4 in both cell types. **d**, *RFD* distribution of FS.

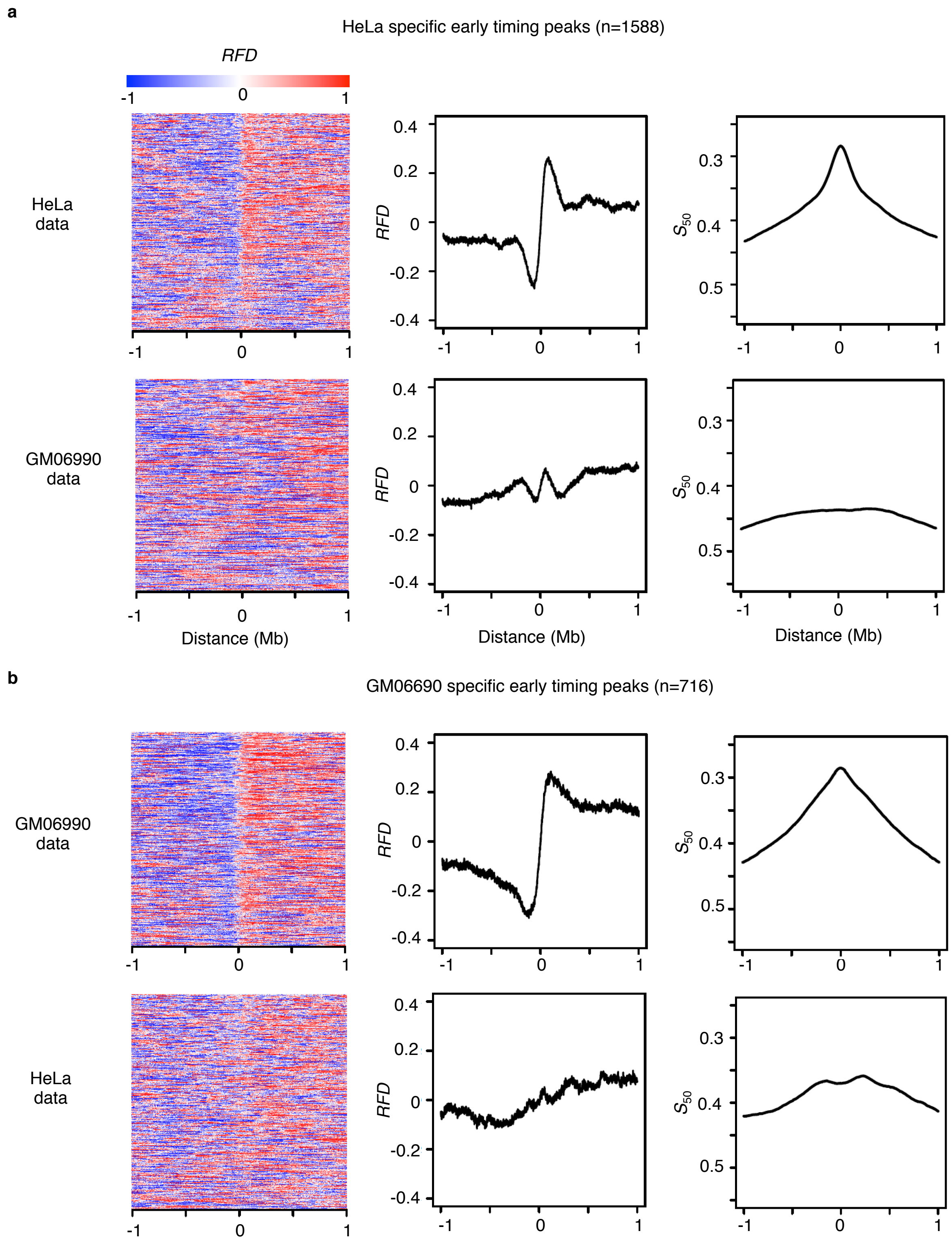

Supplementary Figure 3. *RFD* profiles of cell-type specific, replication timing early peaks. (a), HeLa. **(b),** GM06990. Left, *RFD* heat map around early peaks. Middle, Mean *RFD* profiles. Right, mean S_{50} profiles.

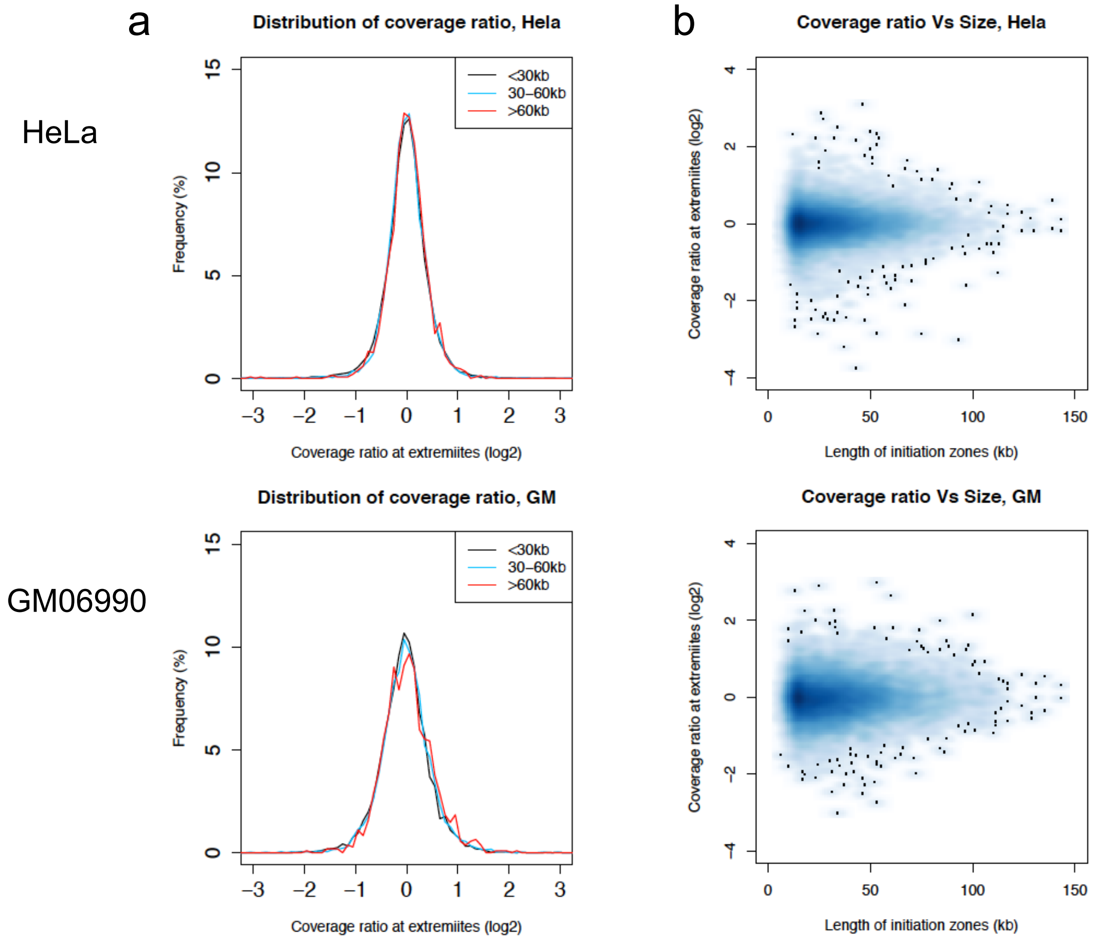

Supplementary Figure 4. The relative coverage of Okazaki fragments at and near AS borders is not related to AS length. In principle, the sum of Crick and Watson ($C+W$) reads should be constant along the genome. If for any reason coverage was low at AS borders, this may artificially broaden the initiation zone due to large fluctuations in ($C-W$)/($C+W$). To investigate this possibility, we computed the ratio of total coverage at each AS border (5kb window) and at the neighboring 5 kb window inside the same AS. The distributions of the $\log_2$(coverage ratio) for AS of indicated size classes **(a)** and the two-dimensional plots of the $\log_2$(coverage ratio) according to AS length **(b)** in the indicated cell lines are symmetric around 0 and therefore show no evidence of biased coverage at borders of AS of any size.

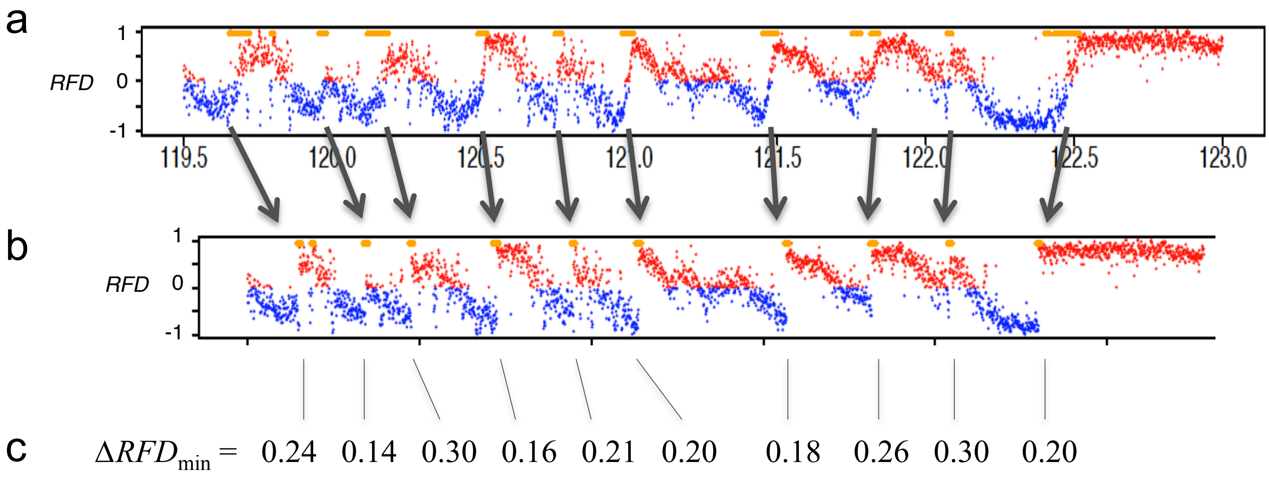

Supplementary Figure 5. Generation and detection of artificial *RFD* shifts mimicking site-specific origins. (**a**) Native *RFD* profile from a 3.5 Mb segment from chromosome 2. Horizontal orange lines indicate HMM-detected AS. (**b**) Artificial *RFD* profile created from (a) by deleting appropriate windows from AS to replace the corresponding smoothly ascending *RFD* segments by abrupt *RFD* upshifts. The HMM-detected AS from this artificial profile are indicated by horizontal orange lines; their lengths all range from 10 kb to 15 kb. Therefore, AS ≤15 kb detected in native *RFD* profiles (1,297 in HeLa and 706 in GM06990) may include site-specific origins. **(c)** For each artificial origin created in (b), the Δ*RFD* between the left and right parts of the upshift was varied down to 10%. The numbers indicate the minimum Δ*RFD* value required for HMM detection.

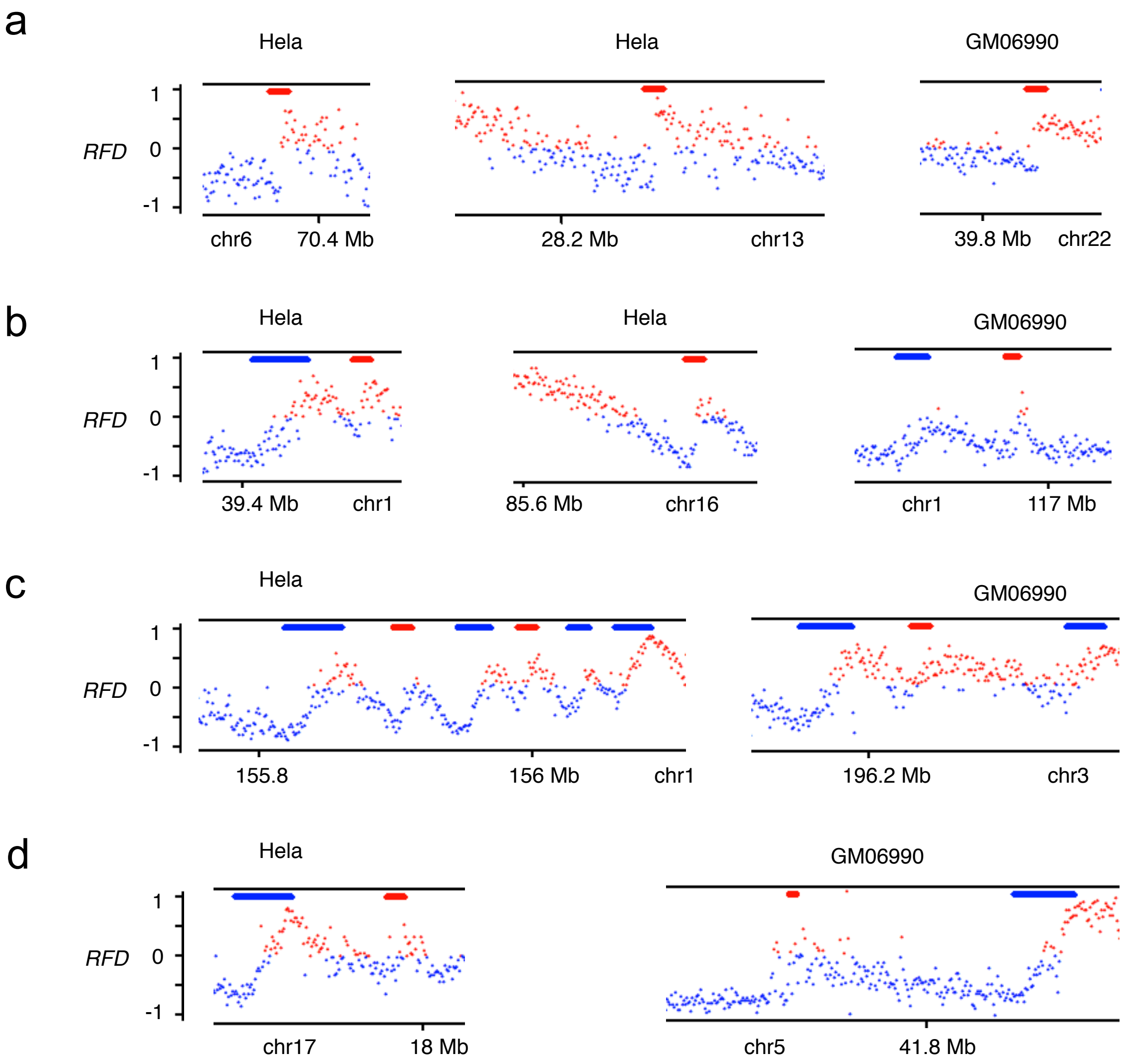

Supplementary Figure 6. Occurence of site-specific origins within AS ≤15 kb from HeLa and GM06990 cells. Examples of **(a)** site-specific (≤1kb) origins ; **(b)** small initiation zones (from 1 kb to 5 kb); **(c)** Broad initiation zones (> 5 kb); **(d)** ambiguous cases. Red and blue horizontal lines indicate AS ≤ 15 kb and AS > 15 kb, respectively. In (a) and (b), the true origin size is smaller than the detected AS. In (c), the true origin size is comparable to the detected size. In (d), the profile may be interpreted either as a tandem array of site-specific origins or as a single broad initiation zone with large signal dispersion. The numbers of site-specific origins are presented in Supplementary Table 2. Site-specific origins such as in (a) are only a small minority of all AS ≤15 kb.

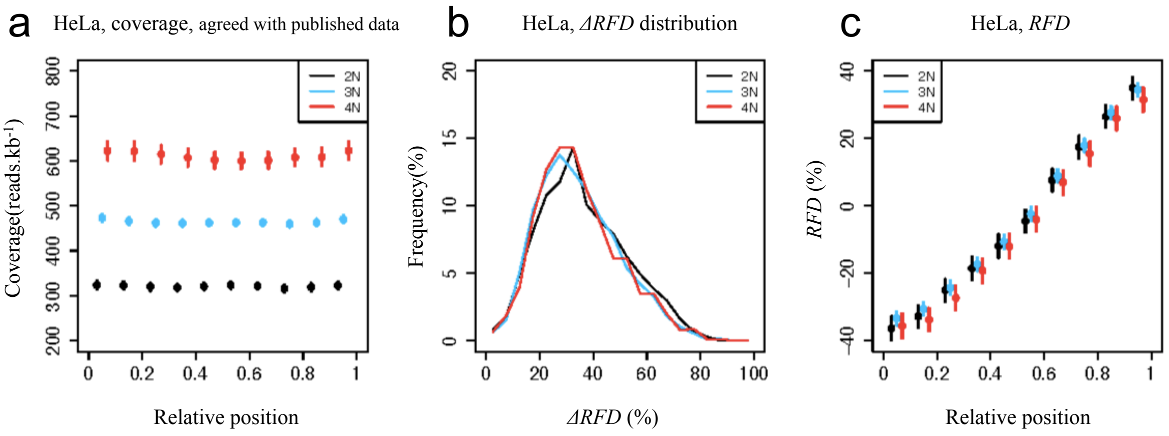

Supplementary Figure 7. The extensive aneuploidy characteristic of HeLa cells does not perturb the behavior of initiation zones. Copy-number profiles of HeLa cells have been reported[7]. **(a)** OK-Seq coverage was in agreement with the reported copy number (dark, N=2; blue, N= 3; red, N=4) and constant throughout initiation zones. **(b)** The distribution of initiation zone efficiencies (*ΔRFD*) was similar for initiation zones whose copy number is 2, 3 or 4. **(c)** The *RFD* changed in a similar linear manner across initiation zones whose copy number is 2, 3 or 4.

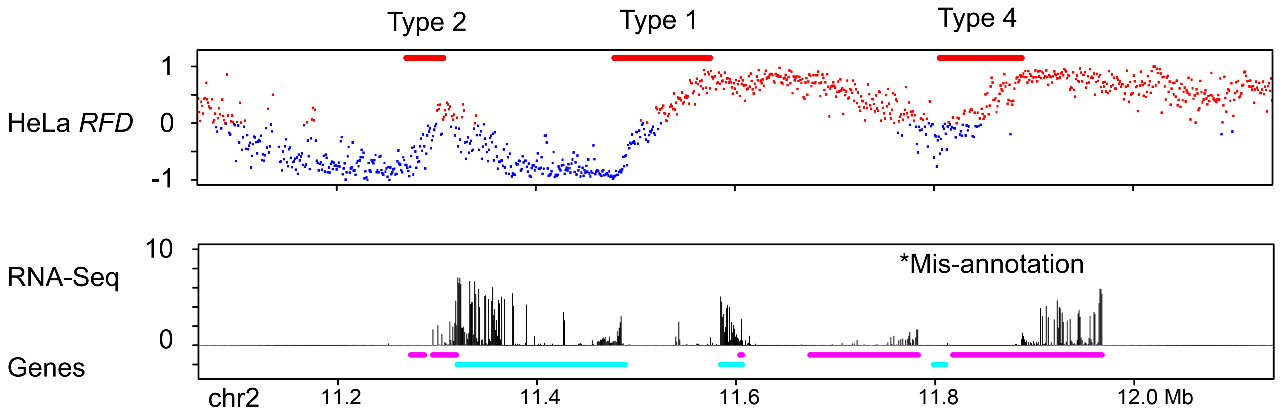

Supplementary Figure 8. *RFD* profile showing examples of type 1, type 2 and type 4 AS. The RNA-Seq profile suggests that in HeLa cells the gene covering the type 4 AS is in fact only transcribed outside the AS. This segment is therefore a type 1 AS.

a

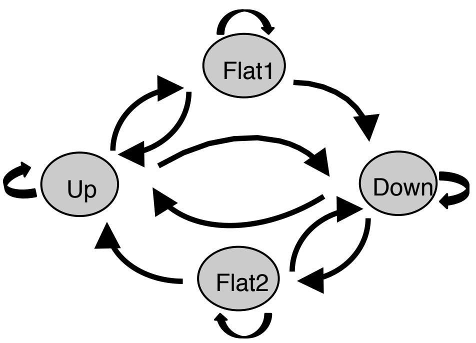

b Emission probabilities

	1st	2nd	3rd	4th	5th
**Down**	0.38388626	0.25592417	0.17061611	0.11374410	0.07582938
**Flat1**	0.10000000	0.20000000	0.40000000	0.2000000	0.10000000
**Flat2**	0.10000000	0.20000000	0.40000000	0.2000000	0.10000000
**Up**	0.02222222	0.03333333	0.06666667	0.21111111	0.66666667

c Transition probabilities

From / to	Down	Flat 1	Flat 2	Up
**Down**	0.99990	0.00000	0.00002	0.00008
**Flat 1**	0.00003	0.99900	0.00000	0.00097
**Flat 2**	0.00097	0.00000	0.99900	0.00003
**Up**	0.00008	0.00002	0.00000	0.99990

d HeLa ascending segments

Init.	Type 1	Type 2	Type 3	Others	Total
**S1**	1,592	1,402	384	1,150	4,528
**S2**	237	819	760	495	2,311
**S3**	42	310	974	187	1,513
**S4**	3	101	1,281	95	1,480
**Total**	1,874	2,632	3,399	1,927	9,832

e GM06990 ascending segments

Init.	Type 1	Type 2	Type 3	Others	Total
**S1**	916	611	115	544	2,186
**S2**	252	474	180	286	1,192
**S3**	89	347	291	231	985
**S4**	12	147	1,039	147	1,345
**Total**	1,269	1,579	1,625	1,208	5,681

f HeLa descending segments

Ter.	Majority Transcribed regions	Majority Non-transcribed regions	Total
**S1**	2,619	1,030	3,649
**S2**	1,158	1,313	2,471
**S3**	414	1,261	1,675
**S4**	135	1,510	1,645
**Total**	4,326	5,114	9,440

g GM06990 descending segments

Ter.	Majority Transcribed regions	Majority Non-transcribed regions	Total
**S1**	1,211	485	1,696
**S2**	646	553	1,199
**S3**	448	748	1,196
**S4**	231	1,393	1,624
**Total**	2,536	3,179	5,715

h HeLa high |*RFD*| flat segments

Ter.	Majority Transcribed regions		Majority Non-transcribed regions	Total
	Co-oriented	Counter-oriented		
**S1**	172	26	72	270
**S2**	105	20	134	259
**S3**	19	1	53	73
**S4**	3	0	12	15
**Total**	299	47	271	617

i GM06990 high |*RFD*| flat segments

Ter.	Majority Transcribed regions		Majority Non-transcribed regions	Total
	Co-oriented	Counter-oriented		
**S1**	170	61	156	387
**S2**	102	23	149	274
**S3**	47	10	106	163
**S4**	9	0	16	25
**Total**	328	94	427	849

Supplementary Figure 9. HMM detection of *RFD* profile segments. a, state model used in the HMM segmentation (Methods); Up, regions of predominant initiation, i.e. AS; Down, regions of predominant termination, i.e. DS; Flat1 and Flat2, FS. **b, c**, emission (**b**) and transition (**c**) probabilities. Numbers of HMM-detected ascending (**d, e**); descending (**f, g**); or flat segments with |*RFD*| > 0.6 (**h, i**) in HeLa (**d, f, h**) or GM06990 (**e, g, h**) *RFD* profiles in successive periods of S phase (S1-S4).

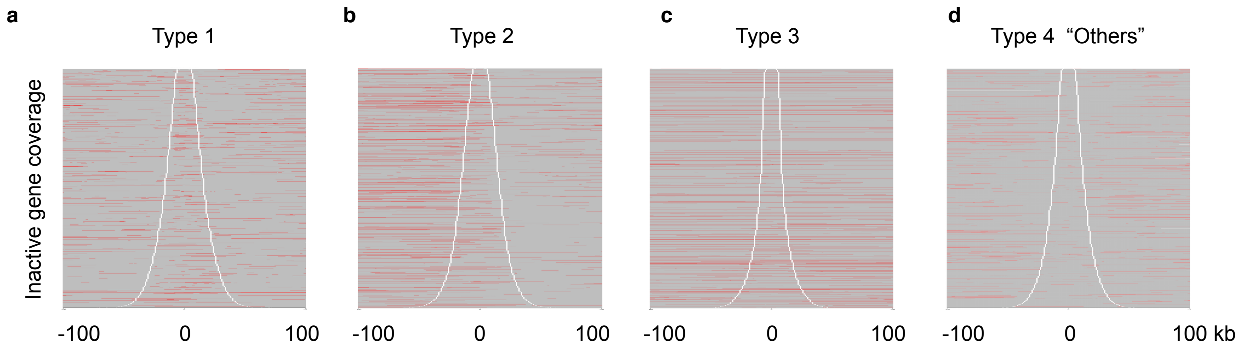

Supplementary Figure 10. Inactive gene coverage around AS. Inactive genes (red) around HeLa type 1 (**a**), type 2 (**b**), type 3 (**c**) and type 4 (**d**) AS, ordered by length and centred at zero abscissa.

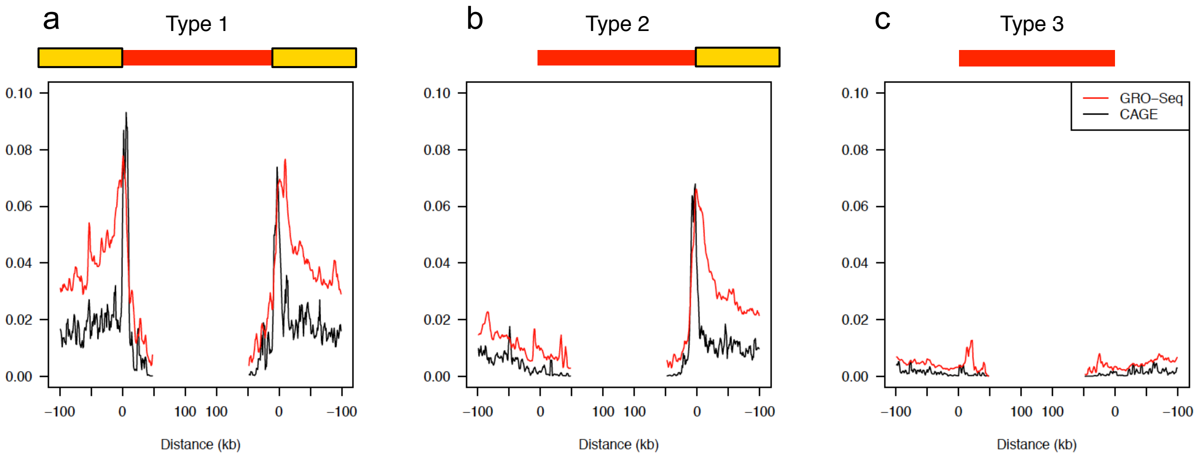

Supplementary Figure 11. Non-coding transcript abundance around AS. Densities of GRO-Seq (orange) and CAGE (black) transcripts[8] around HeLa type 1 **(a)**, type 2 **(b)** and type 3 **(c)** AS.

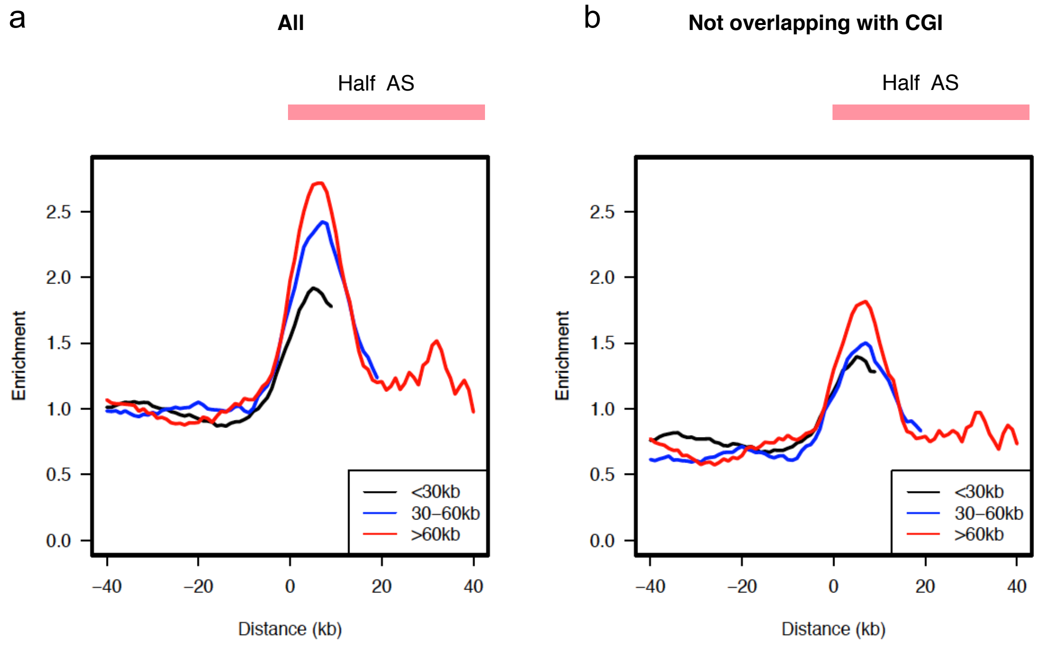

Supplementary Figure 12. Enrichment ratios of ORC1 around borders of AS of different sizes. Enrichment ratios of ORC1 (HeLa) around AS borders (HeLa), computed as in Fig. 6b but for the indicated size classes, for all ORC1 peaks **(a)** or for ORC1 peaks not overlapping CGIs **(b)**.

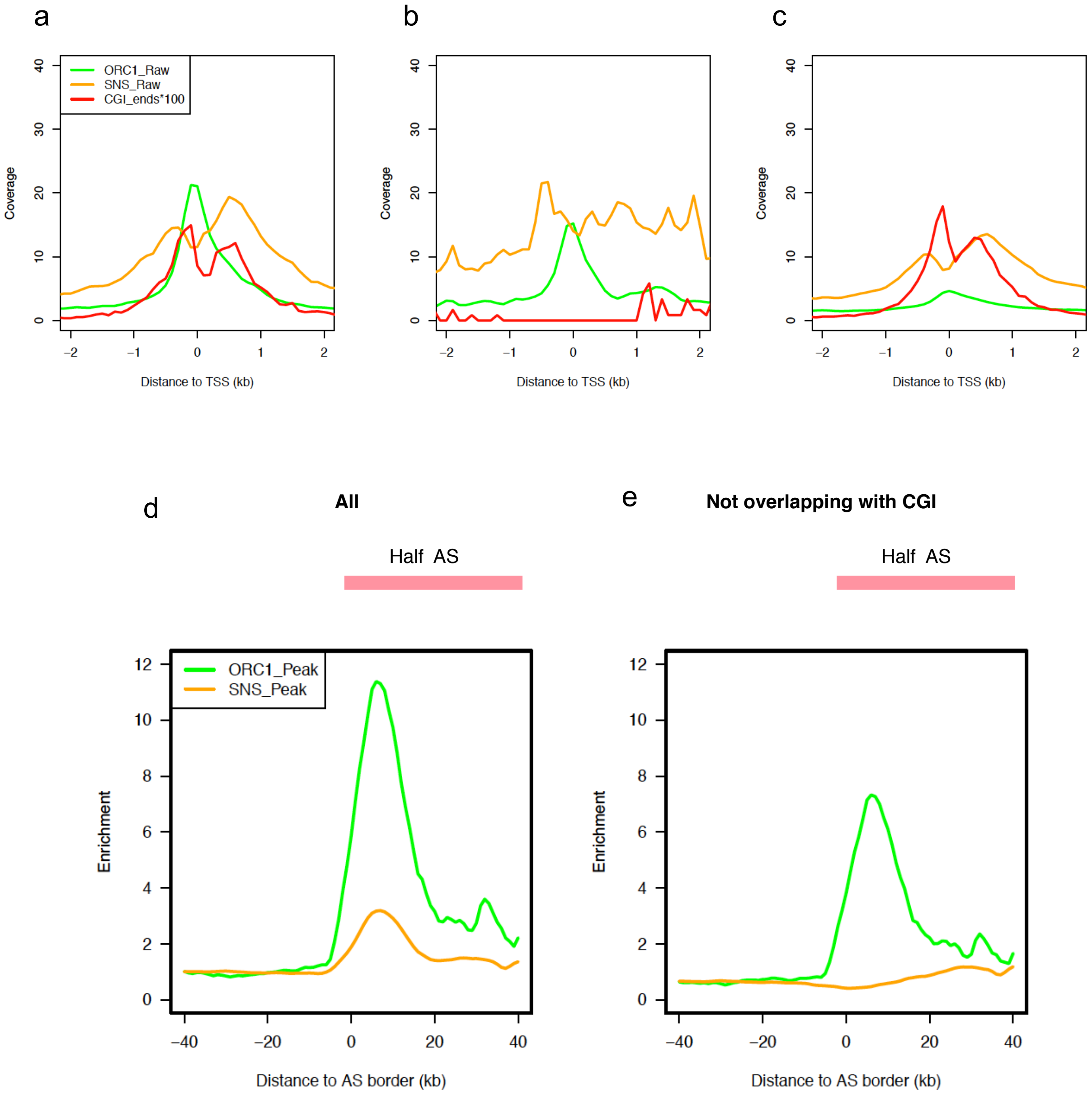

Supplementary Figure 13. Enrichment ratios of ORC1, SNS and CGIs around HeLa TSSs positive for both ORC1 and SNS. (a) Bimodal enrichment of SNS and CGI ends around a single ORC1 peak for the 2,584 HeLa TSSs that are associated with at least one ORC1 peak (within <1 kb) and at least one SNS peak (within <1kb). Among these, 2,464 are associated with a CGI. **(b)** The bimodal SNS pattern disappears, but the single ORC1 peak persists, around the 120 TSSs that are not associated with CGIs. **(c)** A similar bimodal enrichment of SNS and CGIs is observed around the 9,134 HeLa TSSs that are associated with CGIs but not with ORC1. Therefore, the bimodal enrichment pattern of SNS is due to association of SNS with CGIs but not with ORC1. **(d-e)** Enrichment ratios of ORC1 (HeLa) around the 1,635 HeLa AS that overlap (> 1 nt) ORC1 and SNS peaks and whose border(s) is (are) associated with a TSS (within < 20 kb), computed as in Fig. 6b for all peaks **(d)** or for peaks not overlapping CGIs **(e)**. Therefore, SNS are associated with CGIs but not with ORC1 at the borders of AS that are positive for both ORC1 and SNS peaks.

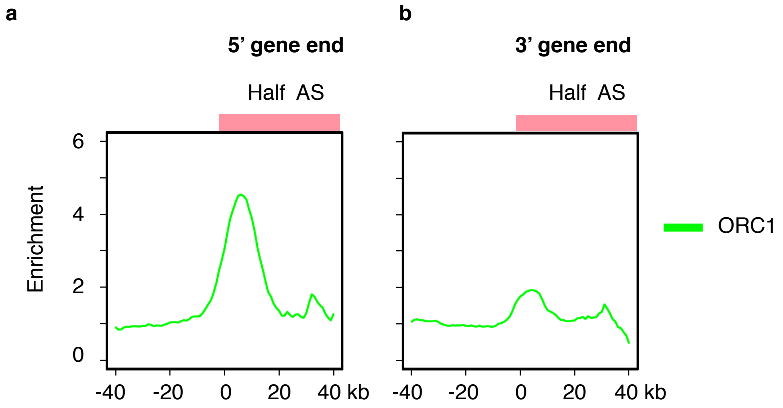

Supplementary Figure 14. Enrichment ratios of ORC1 peaks around AS borders associated with 5’ or 3’ gene ends. Enrichment ratios of ORC1 (HeLa, green) peaks around HeLa AS borders, computed as in Fig. 6b, for AS associated with 5’ (**a**) or 3’ (**b**) gene ends.

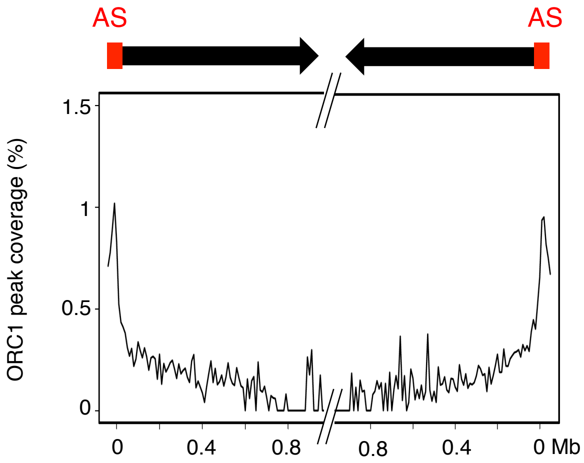

Supplementary Figure 15. Percentage of ORC1 peak coverage (10 kb bin) between consecutive Hela AS.

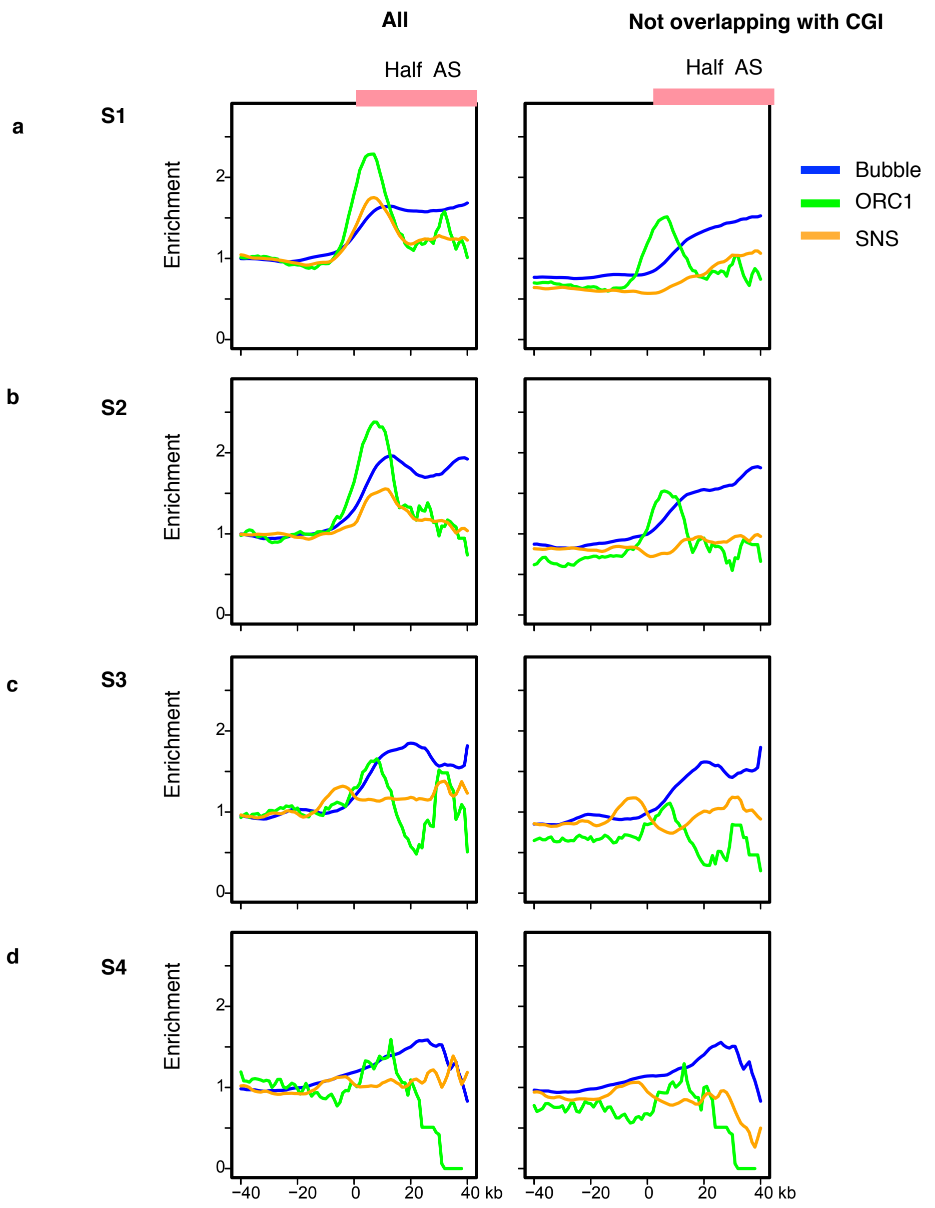

Supplementary Figure 16. Enrichment ratios of Bubble, ORC1 and SNS peaks around borders of ascending segments at different periods of S phase. (a-d), Enrichment ratios of bubbles (GM06990, blue), ORC1 (HeLa, green) and SNS (HeLa, yellow) peaks around AS borders of the same cell type, computed as in Fig. 6b, for AS with mean replication timing within S1 (**a**), S2 (**b**), S3 (**c**) and S4 (**d**).

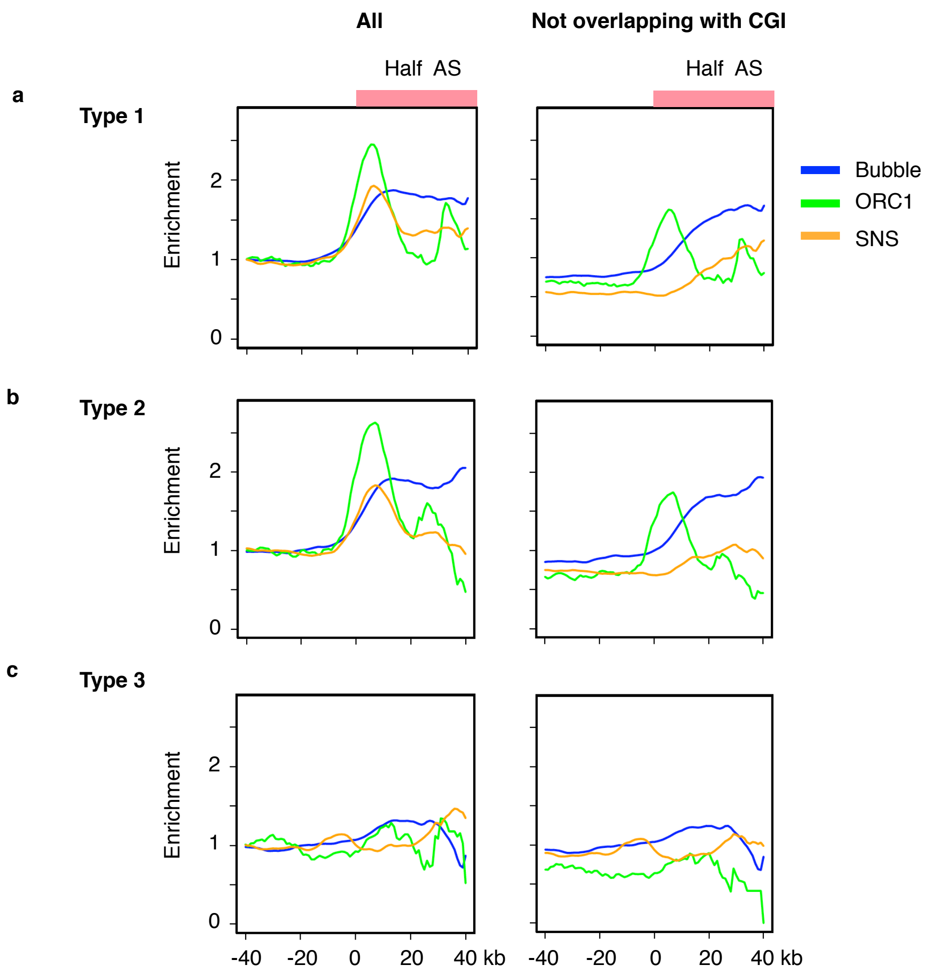

Supplementary Figure 17. Enrichment ratios of Bubble, ORC1 and SNS peaks around borders of AS of different type. Enrichment ratios of bubbles (GM06990, blue), ORC1 (HeLa, green) and SNS (HeLa, yellow) peaks around AS borders of the same cell type, computed as in Fig. 6b, for type 1 (**a**), type 2 (**b**) and type 3 (**c**) AS .

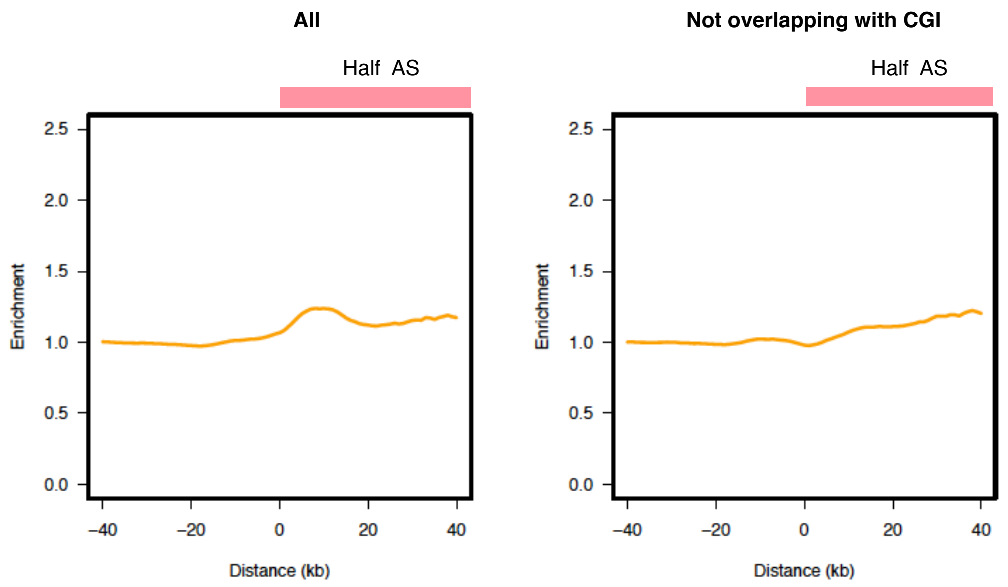

Supplementary Figure 18. Enrichment ratios of SNS raw data[9] around borders of HeLa AS. Enrichment ratios were computed as in Fig. 6b.

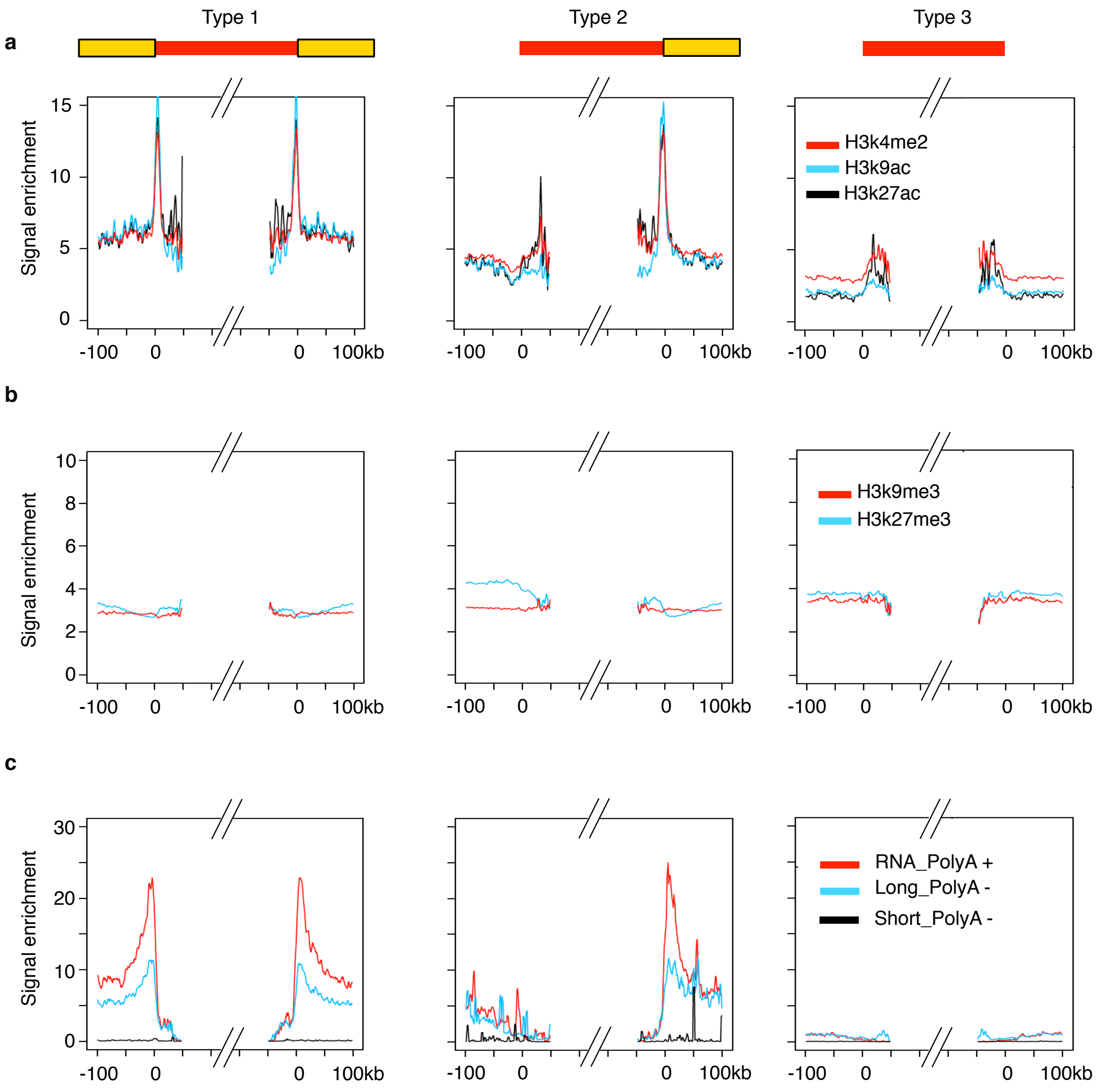

Supplementary Figure 19. Density of histone marks and RNA abundance around AS. (a,b), densities of signal enrichment of indicated histone marks around the HeLa type 1, type 2 and type 3 AS as in Fig. 7. **(c)**, Densities of indicated RNAs around the HeLa type 1, type 2 and type 3 AS; RNA-Seq data were retrieved from Encode[4].

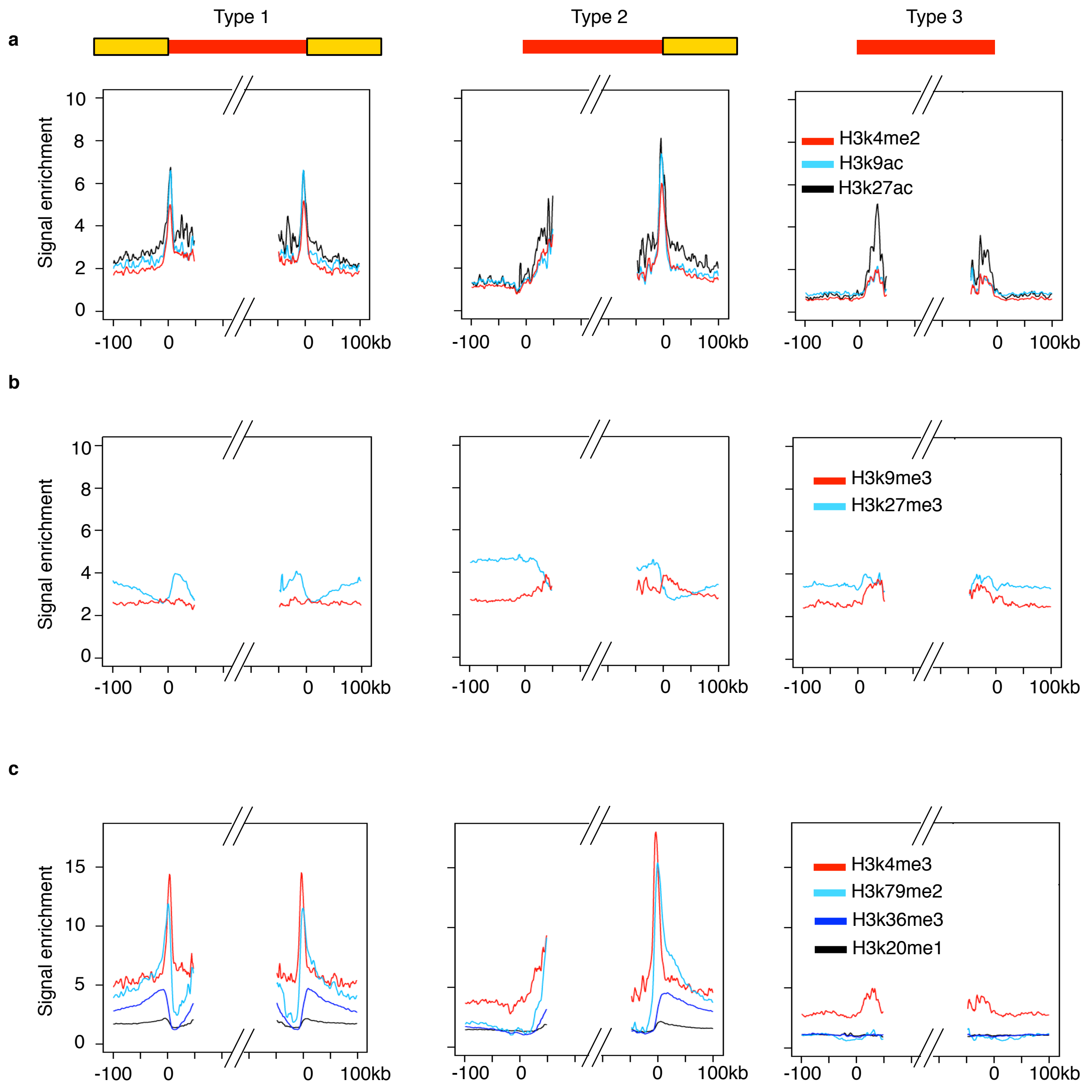

Supplementary Figure 20. Density of histone marks around GM06990 AS . (a-c), densities of signal enrichment of indicated histone marks around the GM06990 type 1, type 2 and type 3 AS as in Fig. 7.

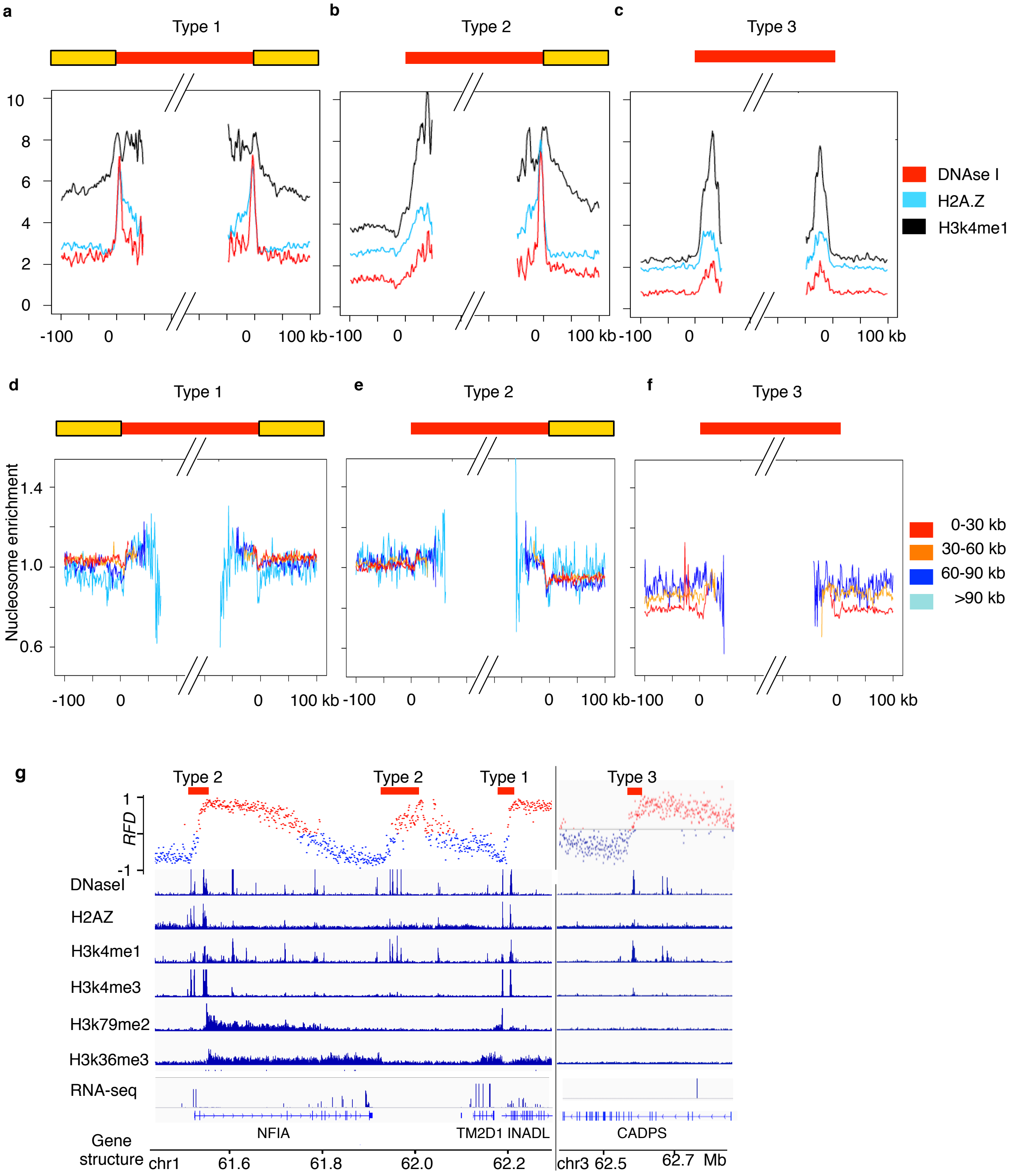

Supplementary Figure 21. Density of open histone marks and DNAse I HSS and nucleosomes around AS. (a-c), Same as in Fig. 7d-f for type 1 (**a**), type 2 (**b**) and type 3 (**c**) AS of GM06990 cells. **(d-f)**, nucleosome enrichment profiles around type 1 (**d**), type 2 (**e**) and type 3 (**f**) AS of GM06990 cells with size 0-30 kb (red), 30-60 kb (yellow), 60-90 kb (blue) and >90 kb (light blue); the nucleosome enrichment profiles detected by MNase-Seq were retrieved from Encode[4]. (**g**), Exemplary HeLa regions showing enrichment of Dnase I HSS, H2A.Z and H3K4me1 within AS (red lines).

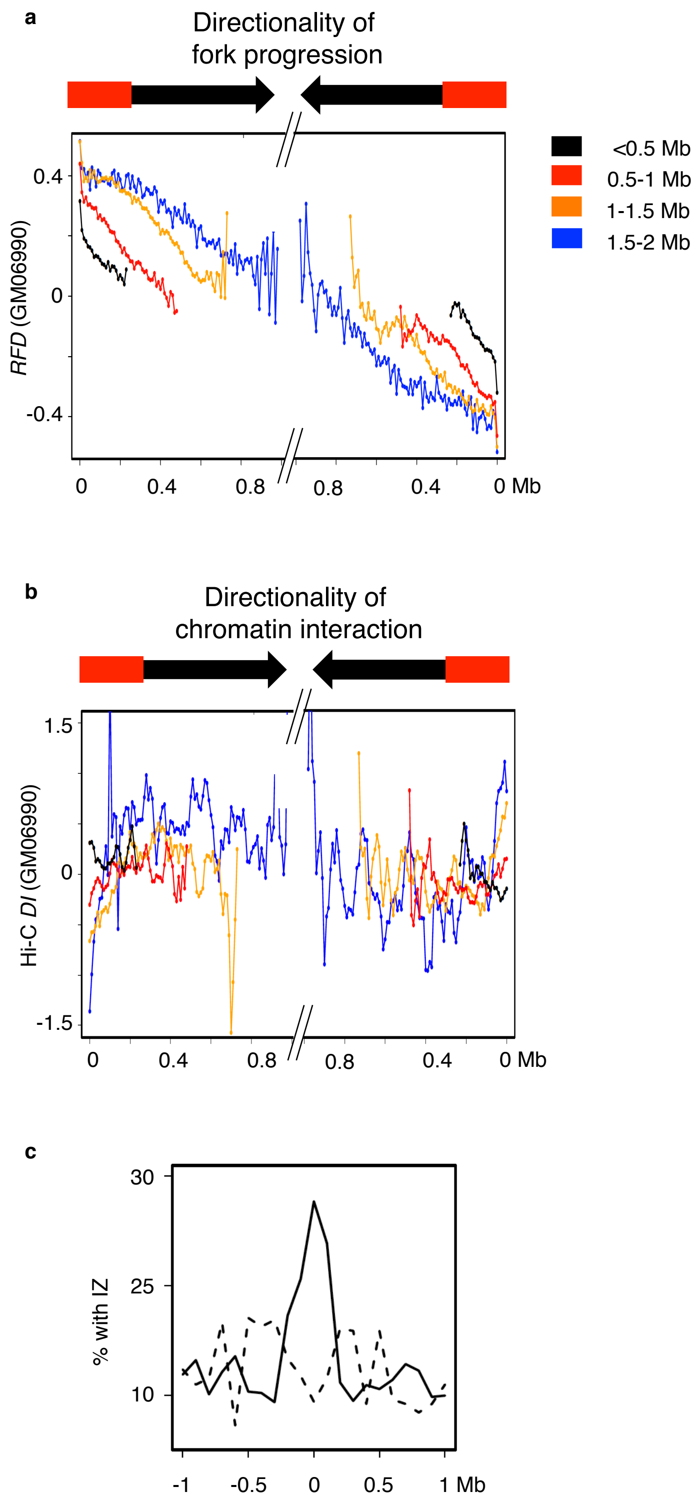

Supplementary Figure 22. (a,b) Co-orientation of replication fork **(a)** and chromatin interaction **(b)** directionalities between consecutive GM06990 AS, as in Fig. 7g for HeLa cells. **(c)** Percentage of genomic segments (100 kb bin) overlap with GM06990 AS around the borders (line) or centres (dash) of TADs shared by IMR90 and H1ESC, as in Fig. 7h for HeLa AS.

Ascending segments (AS)									
	Common			HeLa Specific			GM06990 Specific		
	Number	Mean length (kb)	Genome Coverage (%)	Number	Mean Length (kb)	Genome Coverage (%)	Number	Mean Length (kb)	Genome Coverage (%)
**All**	4186 (4111)	33 (34)	5,2 (5,3)	5650	30	6,3	1573	32	1,9
**CGI -**	1710 (1707)	25 (26)	1,6 (1,6)	3416	26	3,3	1039	28	1,1
**CGI +**	2476 (2404)	39 (40)	3,6 (3,6)	2234	35	2,9	534	38	0,8
**Type 1**	1078 (1085)	39 (41)	1,6 (1,7)	796	34	1,0	184	41	0,3
**Type 2**	1222 (1188)	38 (39)	1,7 (1,7)	1408	35	1,8	389	36	0,5
**Type 3**	1094 (1003)	23 (22)	0,9 (0,8)	2305	26	2,2	622	26	0,6
**Type 4**	792 (835)	33 (35)	1,0 (1,1)	1141	29	1,2	378	33	0,5
**S1**	2136 (1747)	37 (40)	2,9 (2,6)	2392	33	2,9	439	40	0,7
**S2**	881 (856)	35 (37)	1,1 (1,2)	1430	31	1,6	336	32	0,4
**S3**	594 (619)	28 (32)	0,6 (0,7)	919	27	0,9	339	32	0,4
**S4**	573 (887)	22 (22)	0,5 (0,7)	907	24	0,8	458	23	0,4

Descending segments (DS)									
	Common			HeLa Specific			GM06990 Specific		
	Number	Mean length (kb)	Genome Coverage (%)	Number	Mean Length (kb)	Genome Coverage (%)	Number	Mean Length (kb)	Genome Coverage (%)
**All**	5788 (4826)	163 (192)	35,1 (34,6)	3653	106	14,4	889	128	4,3
**S1**	2121 (1559)	103 (135)	8,1 (7,8)	1528	71	4,0	137	79	0,4
**S2**	1581 (1031)	169 (179)	10 (6,9)	890	119	4,0	168	110	0,7
**S3**	1201 (1003)	197 (219)	7,5 (8,2)	654	136	3,3	193	113	0,8
**S4**	1065 (1233)	240 (255)	9,5 (11,7)	580	145	3,1	391	161	2,3

High |*RFD*| flat segments (FS)									
	Common			HeLa Specific			GM06990 Specific		
	Number	Mean length (kb)	Genome Coverage (%)	Number	Mean Length (kb)	Genome Coverage (%)	Number	Mean Length (kb)	Genome Coverage (%)
**All**	236 (235)	193 (306)	1,7 (2,7)	381	172	2,4	614	245	5,6
**S1**	102 (98)	133 (240)	0,5 (0,9)	168	105	0,7	289	185	2,0
**S2**	105 (91)	221 (338)	0,9 (1,1)	154	205	1,2	183	279	1,9
**S3**	28 (43)	306 (389)	0,3 (0,6)	45	266	0,4	120	314	1,4
**S4**	1 (3)	108 (321)	<0,1	14	314	0,2	22	378	0,3

Supplementary Table 1. Statistics of common and cell-type specific AS, DS and high |*RFD*| FS from HeLa and GM06990. CGI+ and CGI -, segments associated or not, respectively, with CGIs. Type 1-4 refer to AS type as described in the text. S1-4 refer to the mean replication timing of the segments.

	AS ≤15 kb	site-specific origins (≤1 kb)	small initiation zones (1-5 kb)
**Hela**	1.297	13	53
**GM06990**	706	8	29

Supplementary Table 2. Occurence of site-specific origins within AS ≤15 kb from HeLa and GM06990 cells.

Supplementary References

1. Chen, C. L. *et al.* Impact of replication timing on non-CpG and CpG substitution rates in mammalian genomes. *Genome Res* **20**, 447-457 (2010).
2. Hansen, R. S. *et al.* Sequencing newly replicated DNA reveals widespread plasticity in human replication timing. *Proc Natl Acad Sci U S A* **107**, 139-144 (2010).
3. Gindin, Y., Valenzuela, M. S., Aladjem, M. I., Meltzer, P. S. & Bilke, S. A chromatin structure-based model accurately predicts DNA replication timing in human cells. *Mol Syst Biol* **10**, 722, doi:10.1002/msb.134859 (2014).
4. Djebali, S. *et al.* Landscape of transcription in human cells. *Nature* **489**, 101-108, doi:10.1038/nature11233 (2012).
5. Wang, S., Dijkwel, P. A. & Hamlin, J. L. Lagging-strand, early-labelling, and two-dimensional gel assays suggest multiple potential initiation sites in the Chinese hamster dihydrofolate reductase origin. *Mol Cell Biol* **18**, 39-50 (1998).
6. Demczuk, A. *et al.* Regulation of DNA replication within the immunoglobulin heavy-chain locus during B cell commitment. *PLoS Biol* **10**, e1001360, doi:10.1371/journal.pbio.1001360 (2012).
7. Adey, A. *et al.* The haplotype-resolved genome and epigenome of the aneuploid HeLa cancer cell line. *Nature* **500**, 207-211, doi:10.1038/nature12064 (2013).
8. Andersson, R. *et al.* Nuclear stability and transcriptional directionality separate functionally distinct RNA species. *Nature communications* **5**, 5336, doi:10.1038/ncomms6336 (2014).
9. Besnard, E. *et al.* Unraveling cell type-specific and reprogrammable human replication origin signatures associated with G-quadruplex consensus motifs. *Nat Struct Mol Biol* **19**, 837-844, doi:10.1038/nsmb.2339 (2012).
